# Clustering and control for adaptation uncovers time-warped spike time patterns in cortical networks in vivo

**DOI:** 10.1038/s41598-021-94002-0

**Published:** 2021-07-29

**Authors:** James B. Isbister, Vicente Reyes-Puerta, Jyh-Jang Sun, Illia Horenko, Heiko J. Luhmann

**Affiliations:** 1 Oxford Centre for Theoretical Neuroscience and Artificial Intelligence, Department of Experimental Psychology, University of Oxford, Oxford, UK; 2 The Blue Brain Project, École Polytechnique Fédérale de Lausanne, 1202 Geneva, Switzerland; 3 Institute of Physiology, University Medical Center, Johannes Gutenberg University, Mainz, Germany; 4 NERF, Kapeldreef 75, 3001 Leuven, Belgium; 5 Faculty of Informatics, Universita della Svizzera Italiana, Via G. Buffi 13, 6900 Lugano, Switzerland; 6 imec, Remisebosweg 1, 3001 Leuven, Belgium

**Keywords:** Neural encoding, Sensory processing, Neural circuits, Barrel cortex, Cortex

## Abstract

How information in the nervous system is encoded by patterns of action potentials (i.e. spikes) remains an open question. Multi-neuron patterns of single spikes are a prime candidate for spike time encoding but their temporal variability requires further characterisation. Here we show how known sources of spike count variability affect stimulus-evoked spike time patterns between neurons separated over multiple layers and columns of adult rat somatosensory cortex in vivo. On subsets of trials (clusters) and after controlling for stimulus-response adaptation, spike time differences between pairs of neurons are “time-warped” (compressed/stretched) by trial-to-trial changes in shared excitability, explaining why fixed spike time patterns and noise correlations are seldom reported. We show that predicted cortical state is correlated between groups of 4 neurons, introducing the possibility of spike time pattern modulation by population-wide trial-to-trial changes in excitability (i.e. cortical state). Under the assumption of state-dependent coding, we propose an improved potential encoding capacity.

## Introduction

The neural coding problem addresses how information is encoded by, and decoded from, patterns of action potentials (i.e. spikes) in the nervous system^1–3^. Whether information is encoded by spike counts or spike times, remains the subject of debate and study^4–6^. Responses following sensory stimulation can be analysed to determine principles of neural coding. Precise and reliable temporal spike patterns have been observed over repeated trials of single stimuli^7–12^ and can improve coding (over spike counts) in primary sensory cortices^6,13^. Nevertheless, a large proportion of spike time variability remains unexplained^14^. With the definitive form of spike time encoding undetermined, population spike count (i.e. mean firing rate) encoding is commonly assumed, in which spike count variability averages out over a population^15–18^. Spike time encoding, however, offers the possibility of a fast, efficient and higher capacity coding form, that could leverage the known temporal sensitivity of neural integration^19–21^ and plasticity^22^. This paper aims to better characterise shared spike time variability in the somatosensory cortex over repeated trials of single sensory stimuli, as this constrains the form in which spike times encode sensory information.

**Figure 1:**
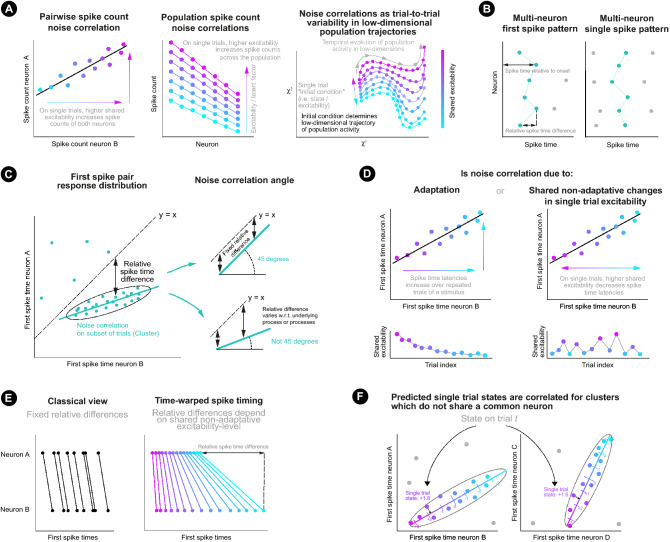
Explanatory figures. (**a**) Illustration of shared spike count variability over repeated trials of a condition for a neuron pair (left), for a population (centre) and exhibited as alternative single trial trajectories through low-dimensional space (right). (**b**) Illustration of multi-neuron first spike patterns (left) and single spike patterns (right). (**c**) Left: Illustrative (artificial) first spike stimulus-response distribution for two neurons and a single stimulus condition. Each point represents the first spike times of the two neurons on a trial on which both neurons spiked. A spike time noise correlation is observable within the isolated cluster. The vertical distance from a point to the line $y=x$ represents the relative spike time difference for the first spike pair. Right: If the angle of the noise correlation is $45^{\circ }$, the relative spike time difference remains fixed (with random noise) w.r.t. the latent process/processes underlying the correlation. If the angle is different from $45^{\circ }$, the relative spike time difference varies w.r.t. the process/processes. (**d**) The latent process underlying the noise correlation may be due to adaptative (left) and/or non-adaptative (right) changes in the shared excitability-level of the two neurons from trial-to-trial. (**e**) Fixed relative spike time differences vs time-warped spike time differences. For the latter, the relative spike time difference is dependent on the non-adaptative single trial shared excitability-level. (**f**) Illustration of correlated predicted cortical state for disjoint pairs of neurons.

As single cortical neurons can generate highly reliable spike trains to direct stimulation in vitro^23^, in vivo response variability largely reflects cortical network dynamics^24^ and synaptic interactions^25,26^. Analysis of response variability shared between neurons over repeated trials of a single stimulus (“noise correlations”) allows the effect of underlying variability sources to be investigated. For spike counts, shared variability may be studied for pairs of neurons and at the population-level^15^ (Fig. 1a, left and centre). The latter includes analysis of latent factors and trial-to-trial variability in the low-dimensional trajectory of population activity^27,28^ (Fig. 1a, right). In the visual cortex, shared spike count variability is modulated by shared trial-to-trial variability in the excitability of neurons across a population. Particularly, neurons across the population respond with either higher or lower spike counts depending on the population excitability level on a single trial (Fig. 1a, centre). Such shared variability has been explained by an additive interaction of stimulus-evoked activity with spontaneous background activity^15,29,30^ and an additional multiplicative interaction^15^. A population’s state at the start of a trial therefore affects the representation of a stimulus. Analysis of population dynamics in the motor cortex also supports the dependence of neural processing on a population’s state at the start of a trial^27,28^ (Fig. 1a, right). Here we characterise how precise spike time representations are modulated by the shared excitability-level of spatially separated neurons on single trials.

Multi-neuron first spike patterns following stimulus onset offer the simplest opportunity to study spike time encoding and are supported as a fundamental form of representation^12,13,31^, which may generalise to multi-neuron single spike patterns (Fig. 1b). For example, the majority of neurons in the barrel field of the rodent somatosensory cortex (“barrel cortex”) respond to single whisker deflections with 0 or 1 spike^12^ and first spike patterns in primary visual cortex are sufficient to enable visual discrimination^31^. With regards to the timing characteristics of multi-neuron single spike patterns, previous analysis of the dataset used here found millisecond precise multi-neuron spike patterns were organised intra- and inter-laminar, as well intra- and inter-columnar in the barrel cortex^12^, and that spike time patterns and (weakly noise correlated) spike counts encoded stimulus information (stimulation frequency and location)^32^. It has also been shown that first spike times can improve decoding of whisker stimulus information by 44% over spike counts^13^. Precise relative differences between the timings of spikes do not require reference to an onset signal to be decoded by downstream neurons (Fig. 1b) and have been observed experimentally^8,9^, including for this dataset^12^. Infrequent reporting of precise relative spike time differences, however, is a key argument against spike time coding. We propose that the temporal variability of spike time patterns requires more detailed characterisation.

The topographic representation of each whisker on the snout of a rodent in the barrel cortex, represents an excellent model to study timing characteristics of multi-neuron single spike patterns in response to controlled sensory stimulation^33^. Using a large dataset of spike sorted recordings in anesthetized adult rat barrel cortex (Methods), this paper aims to characterise the non-trivial timing variability of multi-neuron first spike patterns for groups of neurons separated across layers and columns of the barrel cortex. Firstly, for each stimulus condition and each pair of neurons, the first spike times of the first and second neuron in the pair were plotted against each other for trials of the stimulus condition on which both neurons spiked (Fig. 1c). Within such ‘first spike pair stimulus-response distributions’, spike time clusters (isolated regions of high response probability) were found to be positively correlated above chance-levels. Such positively correlated spike time clusters represent spike time noise correlations (i.e. co-modulation of first spike times by some underlying latent process(es); Fig. 1c).

To the authors’ knowledge, all previous investigations of precise spike time noise correlations have assumed fixed spike time differences, either with a zero^34^ or non-zero^35^ delay, which would correspond with correlation angles of $45^{\circ }$ (Fig. 1c, upper right) in the stimulus-response distribution. In contrast, we find that cluster correlation angles are often different from $45^{\circ }$. This shows that first spike times of neuron pairs co-vary with respect to some underlying latent process(es), such that the relative first-spike time difference varies with respect to this process(es) (Fig. 1c, lower right). This explains why precise fixed relative spike time patterns are seldom reported^8,9,12^.

After controlling for neural stimulus-response adaptation, which causes spike time latencies to increase over rapidly repeated whisker deflections^26,32,36,37^ (Fig. 1d), many clusters remained positively correlated with non-$45^{\circ }$ correlation angles. This cannot be explained by variability in stimulus or cortical onset, which would correspond with a fixed relative difference ($45^{\circ }$ correlation angle; Fig. 1d). Non-$45^{\circ }$ correlation angles suggest that relative first spike time differences are ‘warped’ (i.e. stretched/compressed) depending on the shared excitability-level of the two neurons on a single trial (Fig. 1d,e) and that the shared excitability-level varies from trial-to-trial due to a process other than adaptation. Co-modulation of spike times for neurons separated over multiple cortical columns and layers introduces the poss ibility that spike time patterns are modulated by low-dimensional trial-to-trial population-wide changes in neuronal excitability not caused by adaptation. In support of this (although not conclusive), predictions of single trial excitability were correlated for a large proportion of pairs of clusters, which did not share a common neuron (Fig. 1f). If decoding neurons were also co-modulated by population-wide changes in neuronal excitability, they could integrate spike time patterns in an excitability-dependent (or ‘state-dependent’) manner. An improved potential encoding capacity is predicted under the classical and novel assumptions of ‘fixed-state’ and ‘state-dependent’ decoding, where state defines the shared population excitability-level at the start of a trial.

## Results

Single whisker deflections (sweeps) were made to single whiskers in blocks of typically 200 trials (Methods). For each block, single deflections were made at a certain frequency (between 0.066–10 Hz), such that the inter-trial-interval was 1/f.

**Figure 2:**
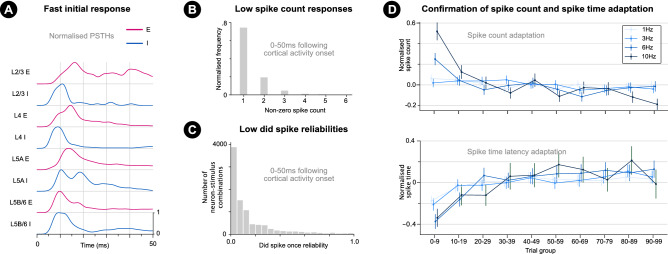
Properties of initial responses of cortical neurons to sensory stimulation. (**a**) Max normalised post-stimulus time histograms (PSTHs) over a 50ms window following stimulus onset (averaged over all trials) for all 8 neuron groups (excitatory in red, inhibitory in blue) in different cortical layers (L) and a 1Hz stimulus. Smoothed with a Gaussian kernel ($\sigma$ = 1.5 ms). (**b**) Normalised spike count histogram over all neurons, trials and stimuli (over 50 ms following cortical activity onset). Zero spike trials are not included. (**c**) Histogram of single neuron single stimulus condition did spike once reliabilities. Each value represents the proportion of trials of a stimulus condition that a single neuron spiked at least once. (**d**) The effect of adaptation over trials (by stimulus-frequency). Top: For each single neuron and stimulus condition combination, spike counts are normalised (mean subtracted followed by division by standard deviation). Means of these normalised spike counts (over single neuron and stimulus condition combinations) are then calculated for stimulation frequency groups (0–0.2, 1, ..., 10 Hz) and 10 trial bins (i.e. trials 0–9, ..., 90–99). These mean values are plotted with a 95% confidence interval ($\pm 2\sigma$) for stimulation frequencies 1, 3, 6, 10 Hz here and the remaining frequencies in Supplementary Fig. 1. Bottom: Same as for spike counts (top) but for normalised first spike times. Data for remaining frequencies shown in Supplementary Fig. 1.

### Confirmation of single spike patterns following stimulus onset

Each neuron-type and layer group demonstrates a clear initial response to single whisker deflections (Fig. 2a). Moreover, the majority of responding neurons spike only once (Fig. 2b), suggesting low spike count encoding capacity. This also supports previous studies^13^ and analyses of the current dataset^12^ in implicating multi-neuron first spike patterns as a prime encoding form. This paper aims to characterise the spike time variability of such multi-neuron single spike patterns. Low response reliability (Fig. 2c) complicates analysis of multi-neuron single spike patterns. For example, a matrix of first spike times (for a single stimulus condition) by neuron and trial index would contain a large number of missing values.

### Confirmation of neuronal stimulus-response adaptation for single neurons

Consistent with previous reports^26,36,37^, including for the current dataset^32^, depressive spike count and spike time latency adaptation were confirmed at stimulation frequencies $\ge 5Hz$ and $\ge 1Hz$ respectively, with the majority of adaptation occurring in the first 20 stimulus trials (Fig. 2d; Supplementary Fig. 1). Such adaptation was confirmed for all neuron groups, excluding spike time adaptation for the undersampled L2/3 I group (Supplementary Fig. 2a). Such spike time latency adaptation can underlie spike time noise correlations and therefore must be controlled for when testing for noise correlations caused by non-adaptative trial-to-trial changes in shared excitability. Controlling for adaptative trends must consider the heterogeneity of responses and adaptation over stimulus trials (Supplementary Fig. 3). Autocorrelation and partial autocorrelation analysis of the the trial-to-trial sequence of first spike times of single neurons supported the presence of general adapatative trends without additional autocorrelative structure in the trial-to-trial sequence of single neuron first spike times (Supplementary Fig. 4).

**Figure 3:**
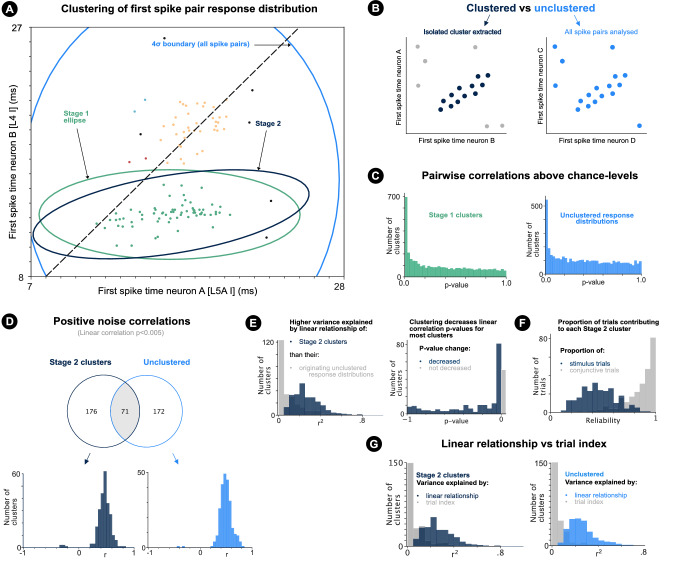
Correlated clusters and correlated unclustered response distributions. (**a**) First spike response distribution for two neurons (L5AI, L4I) over 50ms following cortical onset (1 outlier out of view). Point colours illustrate DBSCAN clustering (epsilon = 1.35; Methods). Green flat ellipse illustrates $4\sigma$ confidence interval of Stage 1 cluster. Dark blue angled ellipse illustrates $4\sigma$ confidence interval of Stage 2 cluster. Light blue ellipse illustrates $4\sigma$ ellipse of all spike pairs under independence and normality assumption. (**b**) Illustration of detected cluster in first spike pair response distribution (left) and unclustered first spike pair response distribution (right). (**c**) Linear regression *p*-values for Stage 1 clusters (left) and unclustered response distributions (right). (**d**) Top: number of correlations $(p<0.005)$ detected for the clustered (Stage 2 clusters) and unclustered case. Intersection shows the number of response distributions from which a correlation was detected by the clustering algorithm and for the unclustered case. Bottom: linear regression r-values of correlated $(p<0.005)$ Stage 2 clusters (left) and unclustered response distributions (right). (**e**) Left: histogram of variance explained ($r^2$) by the spike time linear relationship of each Stage 2 cluster (dark blue) and the linear relationship of the unclustered response distributions from which Stage 2 clusters were extracted (grey). Right: histogram of change in linear regression *p*-values introduced by clustering for response distributions from which Stage 2 clusters were extracted. Particularly, histogram shows Stage 2 cluster linear regression *p*-values minus originating response distribution linear regression *p*-values. Clustering reduces *p*-values in most cases (dark blue). (**f**) Histogram showing for each Stage 2 cluster, the proportion of conjunctive (both neurons spike) trials contributing a sample to the cluster (grey) and the proportion of stimulus trials contributing a sample to the cluster (dark blue). (**g**) Histogram of $r^2$-values of linear multi-output spike pair prediction from trial index (grey) and linear correlation (dark blue, blue) for Stage 2 cluster spike times (left) and unclustered response distributions (right).

### Detection of spike time noise correlations

Analysing the joint responses of two neurons allows noise correlations caused by adaptation and non-adaptative trial-to-trial changes in shared excitability to be discerned. The first spike times of pairs of neurons were plotted against each other for trials of a single stimulus condition on which both neurons spiked. For example, for repeated 4Hz stimulation of a single whisker, Fig. 3a shows the first spike times of two neurons (L5AI and L4I) in the barrel column corresponding to the whisker (i.e. principal column) for trials on which both neurons spiked. First spike pairs appear to come from one of two clusters (isolated regions of high response probability) in the ‘first-spike pair stimulus-response distribution’. Moreover, the cluster of green first spike pairs appears to be correlated with a non-$45^{\circ }$ angle , such that the relative difference between the spike times of the two neurons varies with respect to some underlying latent process(es) (e.g. adaptation or non-adaptative trial-to-trial changes in shared excitability).

Such correlated clusters with apparent non-$45^{\circ }$ angles were observed in many first spike pair distributions (Supplementary Video 1), representing isolated regions of high response probability, which are noise correlated. The occurrence and angle of spike time noise correlations, and the nature of the underlying latent process(es), were quantified both for (i) *isolated clusters* and (ii) the original *unclustered* first spike pair response distributions (Fig. 3b).

For the clustered case, a two-stage algorithm was developed to extract isolated clusters which did not contain smaller isolated sub-clusters. Stage 1 of the algorithm utilised ‘density-based spatial clustering of applications with noise’ (DBSCAN)^38,39^, an established clustering algorithm which detects clusters based on the underlying probability density function. Stage 1 applied two additional steps, firstly discarding DBSCAN clusters, which (like the cluster of orange points in Fig. 3a) were likely to be non-isolated sub-parts of larger clusters, and secondly including points within a flat $4\sigma$ ellipse calculated for the DBSCAN cluster points so that correlation tests were unbiased (Methods).

Stage 1 extracted extracted 3929 well isolated clusters defined by the $4\sigma$ boundary of a flat 2D Gaussian ellipse (Methods; Fig. 3a). Stage 1 did not check for repetitions or similar clusterings produced by different values of the DBSCAN epsilon parameter (to avoid bias). Stage 1 clusters were statistically significantly correlated above chance levels (Fig. 3c, left; Fisher’s method: ${\mathcal {X}}^2$(7858; N = 3929) = 17308:0; *p* < :001) as were the unclustered response distributions (Fig. 3c, right; Fisher’s method: ${\mathcal {X}}^2$(8676; N = 4338) = 14863:4; *p* < :001).

The clustering algorithm found correlated clusters at chance-levels for two surrogate control datasets and was compared to Gaussian Mixture Model approaches (Supplementary Fig. 5; Methods).

An angled ‘Stage 2’ ellipse was calculated to better estimate the distribution underlying each correlated $(p<0.005)$ Stage 1 cluster (Methods; Fig. 3a). Each Stage 2 cluster came from a unique first spike pair response distribution. 247 unique Stage 2 clusters were extracted, 98.0% (242/247) of which remained correlated above chance $(p < 0.005)$.

Unclustered response distributions were also further analysed, and 243 unclustered response distributions were determined as correlated $(p<0.005)$. The detected Stage 2 clusters and correlated unclustered response distributions are shown in (Supplementary Videos 1 and 2 respectively). In the following sections, the same analyses are performed on the correlated Stage 2 clusters and correlated unclustered response distributions. For a subset of first spike pair response distributions, correlations $(p<0.005)$ were detected for both the clustered and unclustered case (Fig. 3d, top).

98.0% (242/247) of Stage 2 clusters and 99.2% (241/243) of correlated unclustered response distributions were positively correlated (Fig. 3d) suggestive of modulation by spike time latency adaptation or trial-to-trial changes in pairwise shared excitability.

Linear relationships explained a large proportion of spike time variance within Stage 2 clusters, but not within the response distributions from which Stage 2 clusters were extracted (Fig. 3e). In fact, clustering was necessary to uncover a large proportion of spike time noise correlations detected through clustering (Fig. 3e). This result partly explains why precise spike time noise correlations are rarely reported. Figure 3f confirms that noise correlated clusters consisted of responses from a subset of stimulus trials.

More variance was explained by the linear relationship between the spike time pairs than by linear models fit to predict the joint spike time pairs from trial number, suggesting that clustered and unclustered noise correlations are modulated more by trial-to-trial changes in pairwise shared excitability than by adaptation (Fig. 3g; tested further below).

### Non-$45^{\circ }$ spike time noise correlation angles

**Figure 4:**
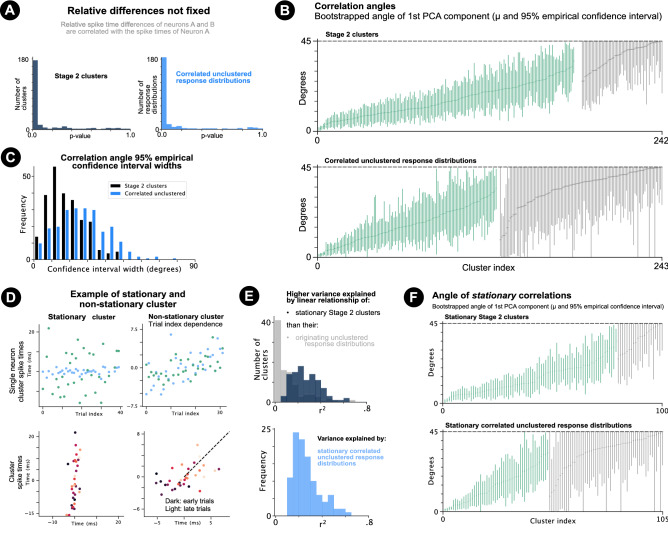
Correlation angles and stationary clusters. (**a**) Histograms of linear regression *p*-values of the first neuron’s spike time and the spike time difference. (**b**) Mean $\theta _{45}$ angles with 95% empirical confidence intervals for positively correlated Stage 2 clusters (top) and unclustered response distributions (bottom). Green if significantly greater than $0^\circ$ (*p*-value < 0.025) and less than $45^\circ$ (*p*-value < 0.025). Grey otherwise. (**c**) Histogram of cluster angle 95% empirical confidence intervals. (**d**) Example of stationary (left) and non-stationary cluster (right) determined by the stationarity criteria (see Methods). Top row shows single neuron cluster spike times for each neuron (green and blue) plotted against cluster trial index. Bottom shows the cluster spike times of neuron A and B plotted against each other. Non-stationary cluster shows a clear trial dependence. (**e**) Top: Histogram of variance explained ($r^2$) by the spike time linear relationship of each stationary Stage 2 cluster (dark blue) and the linear relationship of the unclustered response distributions from which stationary Stage 2 clusters were extracted (grey). Bottom: $r^2$ values shown for stationary unclustered response distributions (**f**) Predicted $\theta _{45}$ angles with 95% empirical confidence intervals for stationary Stage 2 clusters (top) and stationary correlated unclustered response distributions. Angles significantly different from $0^{\circ }$ (*p*-value < 0.025) and $45^{\circ }$ (*p*-value < 0.025) coloured green. Grey otherwise.

For Stage 2 clusters and correlated unclustered response distributions, relative spike time differences were correlated with the spike time of the first neuron in the pair (Fig. 4a). Correlations are therefore not explained by $s_1 = s_0 + d + independent\_noise$ where *d* is a fixed difference and $s_0$ (the spike time of neuron 0) varies from trial-to-trial.

To test whether cluster correlation angles were different from $45^{\circ }$, the correlation angle $\theta$ of each correlated Stage 2 cluster and correlated unclustered response distribution was estimated (defined by the bootstrapped angle that the first principal component made with the positive x-axis; Methods). $\theta _{45} = min(\theta , 90-\theta )$ was used, as only the ordering of the neurons determined whether $\theta$ was above or below $45^{\circ }$. It is important to note that the Henze–Zirkler null hypothesis of normality was rejected $(p < 0.05)$ for 71.7% (177/247) of Stage 2 clusters and 76.5% (186/243) of correlated unclustered response distributions.

73.7% (182/247) and 47.7% (116/243) of $\theta _{45}$ angles were between and significantly different from $0^\circ$ (*p*-value < 0.025) and of $\theta _{45}$ angles were between and significantly different from $0^{\circ }$ (*p*-value < 0.025) and $45^{\circ }$ (*p*-value < 0.025) for Stage 2 clusters and correlated unclustered response distributions respectively (Fig. 4b). This suggests that the relative difference representation of a stimulus is changed by an underlying latent process(es). The clustering algorithm found correlated clusters with correlation angles at $45^{\circ }$ for two additional surrogate control datasets (Supplementary Fig. 6; Methods). The clustering algorithm enabled estimations of correlation angles with smaller confidence intervals compared to the unclustered response distributions (Fig. 4c).

### Stationary correlations

The next sections control for neuronal stimulus-response adaptation to determine when non-$45^{\circ }$ correlations are due to shared trial-to-trial changes in the excitability of neuronal pairs. For Stage 2 clusters and correlated unclustered response distributions, spike times were either not modulated by adaptation, or the cluster spike times of one or both of the neurons were modulated by adaptation (Supplementary Fig. 7). Clusters or response distributions were defined as ‘stationary’ if the spike times of both neurons were not modulated by adaptation, and ‘non-stationary’ otherwise (see Methods stationarity criteria). Figure 4a shows example stationary and non-stationary clusters. The non-stationary cluster shows a clear trial dependence.

40.5% (100/247) of Stage 2 clusters and 43.2% (105/243) of correlated unclustered response distributions were determined as stationary.

Figure 4e shows the proportion of variance explained by the correlations of these stationary Stage 2 clusters and stationary correlated unclustered response distributions.

For stationary Stage 2 clusters, cluster spike times of the first neuron on cluster trials *t*, were not correlated with the cluster spike times of the second neuron on cluster trials $t+1$ (i.e. trial-lag-1 cross-correlation; Supplementary Fig. 8). The same was true for stationary correlated unclustered response distributions. This showed that removal of single trial correlations removed the correlations of the stationary clusters and unclustered response distributions, which would not be the case if general adaptative trends underlied the stationary correlations. Moreover, chance-level autocorrelations and cross-correlations for multiple trial-lags confirmed that the stationary correlations were not affected by adaptative changes or autocorrelative structure (Supplementary Fig. 8), providing an important control test.

77.0% (77/100) of stationary Stage 2 clusters and 43.8% (46/105) of stationary correlated unclustered response distributions had $\theta _{45}$ angles between and significantly different from $0^{\circ }$ and $45^{\circ }$ (Fig. 4f) showing that relative first spike time differences are warped by shared trial-to-trial changes in the excitability of neuronal pairs. It is important to note that the Henze–Zirkler null hypothesis of normality was rejected $(p < 0.05)$ for 75.0% (75/100) of stationary Stage 2 clusters and 76.2% (80/105) of stationary correlated unclustered response distributions.

### Fixed-state and state-conditioned relative difference response distributions

**Figure 5:**
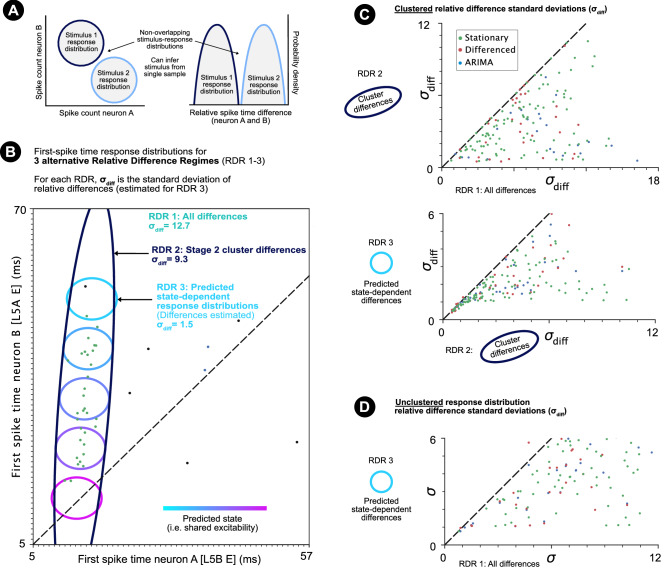
State-dependent coding predictions and relative difference regimes. (**a**) Illustration of two non-overlapping stimulus-response distributions for (left) spike counts and (right) relative spike time differences. (**b**) Sampled first spike response distribution for two neurons (L5B E, L5A E). Point colours illustrate DBSCAN cluster (epsilon = 5.0). Dark blue angled ellipse illustrates $4\sigma$ boundary of Stage 2 ellipse (i.e. the area for which the standard deviation of relative differences is calculated under RDR 2). Coloured circles illustrate the $4\sigma$ boundary of the state-dependent response distribution predicted by factor analysis for single state values ($<0, 0, >0$). The standard deviation of the predicted state-conditioned first spike relative difference distributions (for RDR 3) is calculated based on the size of this area (Methods). (**c**) The standard deviation of differences of RDR 1 vs RDR 2 (top) and RDR 2 vs RDR 3 (bottom) for criteria fulfilling stationary, differenced and ARIMA modelled clusters. (**d**) RDR 2 vs RDR 3 (bottom) for criteria fulfilling stationary, differenced and ARIMA modelled unclustered response distributions.

The paper compares the potential encoding capacity of the characterised clusters under three alternative relative spike time difference coding regimes named Relative Difference Regimes (RDR) 1-3. It is first necessary to consider how downstream neurons discriminate between stimuli. If two sensory stimuli elicit a behaviourally different response, downstream neurons must be able to respond differently to samples from the response distribution of either stimulus. This is possible if the response distributions of the two stimuli are non-overlapping in dimensions that downstream neurons are sensitive to, such as spike counts or relative spike time differences (Fig. 5a). The smaller the region of stimulus-response distribution space that single stimulus-response distributions occupy, the higher the capacity for encoding the presence of different stimuli reliably.

Two possible relative difference coding regimes are firstly considered under the classical assumption of fixed-state-decoding (i.e. decoding neurons are not modulated by processes of adaptation or shared trial-to-trial changes in the excitability of neuronal pairs). Relative Difference Regime 1 (RDR 1) describes the assumption that pairs of neurons encode information through a fixed relative spike time difference. The standard deviation of relative differences over all spike pairs ($RDR1\_\sigma _{diff}$) offers a quantification and prediction of the potential encoding capacity for relative differences under RDR 1. The assumption, however, does not capture the quantified structure of the observed response distributions (Fig. 5b; Supplementary Video 1).

Observation of first spike stimulus-response distributions (Fig. 5b; Supplementary Video 1) shows that clusters correspond to regions of high response probability, which better estimate the underlying stimulus-response distributions. Relative Difference Regime 2 (RDR 2) describes the notion that correlated clusters may represent trials on which the neuron pair’s relative first spike time difference reliably contributes to the representation of the stimulus. Under the classical assumption of fixed-state-decoders, the standard deviation of relative differences ($RDR2\_\sigma _{diff}$) calculated for samples in a stationary Stage 2 cluster quantifies the size of the relative difference response distribution and the potential encoding capacity for relative differences under RDR 2. As stationary clusters are not affected by adaptation, all cluster samples can be used in estimating $RDR2\_\sigma _{diff}$. The smaller first spike pair relative difference standard deviations ($\sigma _{diff}$) under RDR 2 compared to RDR 1 demonstrates an improved potential encoding capacity for RDR 2 (Fig. 5b,c; Supplementary Video 1).

RDR 3 considers that downstream decoding neurons may be modulated by the same shared trial-to-trial changes in the excitability that modulate cluster correlations (i.e. state-dependent-decoding). For example, a higher-level of excitability (i.e. continuous cortical state) on a single trial would decrease the latency of both neurons. Spike times of both neurons would then come from a smaller state-conditioned stimulus-response distribution. If the cluster correlation is different from $45^{\circ }$, as in Fig. 5b, different state values would correspond with different distributions of relative differences. A downstream neuron modulated by the same trial-to-trial changes in excitability (i.e. continuous cortical state) could respond in a state-dependent manner. Such (smaller) state-conditioned first spike difference stimulus-response distributions would provide a higher coding capacity under state-dependent decoding.

To first estimate the size of state-dependent first spike response distributions for each stationary Stage 2 cluster, the following linear single factor model was fit to each zero-centred stationary Stage 2 cluster: $s_i = \lambda _i \eta + \sigma _i$ for $i \in [0, 1]$ (Methods). $\eta$ represents the value of the continuous underlying state (i.e. shared level of excitability) with most values between $[-3, 3]$. $\lambda _i$ is the gradient at which the spike time of neuron *i* varies w.r.t. the state $\eta$, and $\sigma _i$ is the predicted standard deviation of the spike times $s_i$ given $\eta$. Figure 5b illustrates state-dependent first spike response distributions for example values of $\eta = [-2.0, -1.0, 0.0, 1.0, 2.0]$.

The estimated size of state-conditioned distributions of decodable relative spike time differences (RDR 3) was then calculated from the factor analysis model as $\sigma _{diff} = \sqrt{\sigma _{0}^2 + \sigma _{1}^2}$ (see Methods). The estimated standard deviations of state-conditioned distributions of relative differences ($RDR3\_\sigma _{diff}$) offers an improved potential coding capacity over RDR 2 (Fig. 5b,c; Supplementary Video 1) and thus also over RDR 1, but would require confirmation of modulation by a population-wide state, which varies from trial-to-trial. Figure 5d shows the predicted improvement of $RDR3\_\sigma _{diff}$ over RDR 2 for the correlated unclustered response distributions.

### Non-stationary clusters also modulated by shared trial-to-trial changes in the excitability of neuronal pairs

**Figure 6:**
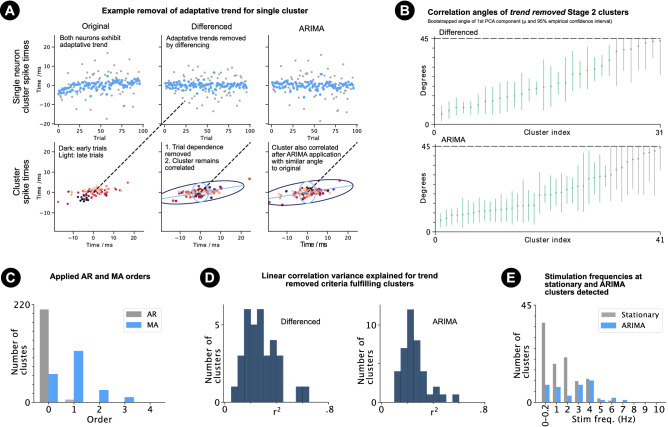
Time series modelling and cluster frequency-dependence. (**a**) For an example cluster, the original spike times do not fulfil the stationarity criteria (Methods) but do after the application of differencing and ARIMA modelling. The factor analysis predicted state-dependent response distributions are therefore presented for the cluster after differencing and ARIMA modelling ($4\sigma$ ellipse; bottom centre and bottom right). It is clear that the adaptative trends have been removed by differening and ARIMA modelling but the clusters remain correlated with similar angles to the original cluster. (**b**) Predicted angles and 95% empirical confidence intervals of differenced and ARIMA modelled criteria fulfilling Stage 2 clusters. Angles significantly different from $0^{\circ }$ and $45^{\circ }$ coloured green. Grey otherwise. (**c**) Order of AR and MA models fit to the single neuron cluster spike times of differenced clusters. (**d**) Linear regression $r^2$ values for criteria fulfilling differenced (left) and ARIMA (right) clusters. (**e**) Histogram showing number of stationary Stage 2 clusters and criteria fulfilling ARIMA modelled clusters (by stimulation frequency).

To test whether shared non-adaptative trial-to-trial changes in the excitability of neuronal pairs also modulate the remaining non-stationary Stage 2 clusters and non-stationary correlated unclustered response distributions, and to estimate the size of underlying relative difference response distributions at a single point in time under RDR 2 and 3, single neuron adaptative trends of non-stationary clustered and unclustered spike times were removed using time series modelling. Two techniques with different advantages were applied to non-stationary spike times (Fig. 6a). For the clustered case, adaptative trends were not removed from spike times before clustering so as to ensure that clusters and spike time correlations were not introduced by such pre-processing.

Simple parametric models were not used to remove trends because non-stationarities in the trial-to-trial sequence of single neuron and cluster spike time did not always follow standard adaptative/non-stationary trends (Supplementary Fig. 8-3; Methods). In these cases, standard parameteric models would not remove the non-stationarity and would risk introducing correlations were none where present.

Firstly, first order ‘differencing’^40^ was applied to remove adaptative trends of single neuron spike times determined as non-stationary by the stationarity criteria (Fig. 6a; Methods). Particularly, spike times on trials *t* were subtracted from spike times on trials $t+1$ (for trials that contributed samples to the cluster or unclustered response distribution). As differencing increases the standard deviation of the time series, the differenced time series were rescaled appropriately (Methods). Negative autocorrelations known and shown here to be introduced by differencing (Supplementary Fig. 9) were ignored, because the resulting spike times allowed direct assessment of correlation angles after trend removal without further processing.

76.2% (112/147) of non-stationary clusters and 80.4% (111/138) of non-stationary correlated unclustered response distributions had differencing applied to at least one neuron. After differencing, 29.5% (33/112) and 33.3% (37/111) of these clusters and unclustered response distributions fulfilled the stationarity and correlation criteria (see Methods), of which 72.7% (24/33) and 54.1% (20/37) had $\theta _{45}$ angles between and significantly different from $0^{\circ }$ (*p*-value < 0.025) and less than $45^{\circ }$ (*p*-value < 0.025; Fig. 5b). It is important to note that the Henze–Zirkler null hypothesis of normality was rejected $(p < 0.05)$ for 21.2% (7/33) and 62.2% (23/37) of the criteria fulfilling differenced Stage 2 clusters and unclustered response distributions.

The second technique fitted autoregressive (AR) and/or moving average (MA) models appropriately to the differenced time series (i.e. ‘ARIMA’ modelling^41,42^), in order to remove the autocorrelations present in the time series and/or introduced by differencing, and to return the time series to the original scale without rescaling (Methods). The ’I’ in ARIMA refers to the differencing applied previously in the first step. The AR model component regresses the values of the time series on values of the time series on previous trials, where the time series is a trial-to-trial sequence of first spike times. The MA model component regresses the value of a time series on a moving average of the previous values. The order of the AR and MA components determines the number of previous trial first spike times that are used. An order of 0 means that no AR or MA component was applied. Orders were selected automatically (Methods).

96.4% (108/112) and 100.0% (111/111) of these differenced clusters and differenced unclustered response distributions had an AR and/or MA model applied to at least one neuron.

The majority of fitted AR or MA models were of the MA type with a trial lag of 1 (Fig. 6c, Supplementary 9-3), as expected for removal of the negative autocorrelations introduced by differencing. That is, the algorithm automatically selects to remove the dependence of a spike time on the previous spiking trial’s spike time (a dependence that is introduced by the differencing operation).

37.0% (40/108) and 39.6% (44/111) of ARIMA modelled clusters and unclustered response distributions fulfilled the criteria, of which 72.5% (29/40) and 56.8% (25/44) had $\theta _{45}$ angles between and significantly different from $0^{\circ }$ (*p*-value < 0.025) and less than $45^{\circ }$ (*p*-value < 0.025; Fig. 6b, Supplementary Fig. 9-3). The Henze–Zirkler null hypothesis of normality was rejected $(p < 0.05)$ for 21.2% (7/33) and 62.2% (23/37) of the criteria fulfilling ARIMA modelled Stage 2 clusters and unclustered response distributions. Autocorrelations and cross correlations were below chance levels for the criteria fulfilling ARIMA modelled clusters (Supplementary Fig. 10), showing that AR and MA modelling successfully removed the negative autocorrelations introduced by differencing.

These results demonstrated that the spike times and relative differences of a proportion of non-stationary Stage 2 clusters and unclustered response distributions are also modulated by shared non-adaptative trial-to-trial changes in the excitability of neuronal pairs. Figure 6d and Supplementary Fig. 9-3 show the variance explained by the correlations after trend removal (differencing and ARIMA). The angles of the differenced and ARIMA modelled spike times corresponded with the correlation angles before the application of differencing and ARIMA modelling (Supplementary Fig. 11). This supports co-modulation by shared non-adaptative trial-to-trial changes in the excitability of neuronal pairs and shows that the non-$45^{\circ }$ correlation angles are not artifacts of time series analysis.

The trend removed clusters allowed all cluster samples to be used in the estimation of the underlying cluster and corresponding distribution of relative differences (RDR 2) at a single point in time. Factor analysis was applied to the trend removed clusters to estimate state-dependent distributions of relative differences at a single point in time (RDR 3). Again, RDR 2 gave an improved potential encoding capacity over RDR 1, whilst RDR 3 gave an improved potential encoding capacity over both RDR 1 and 2 (Fig. 6c,d).

### Frequency dependence and spatial distribution of correlated clusters

Stationary correlations were found at all stimulation frequencies, but mostly at lower frequencies (Fig. 6e, Supplementary Fig. 9-3), showing that lower frequencies are better suited for stimulus response distribution characterisation as adaptation occurs to a lesser extent. ARIMA modelled clusters were found for all frequencies $\le 8Hz$ (Fig. 6e, Supplementary Fig. 9-3), demonstrating that the techniques can characterise noise correlations caused by shared non-adaptative trial-to-trial changes in the excitability of neuronal pairs in the presence of adaptation and non-stationarity, which occur both at lower frequencies suited for response distribution characterisation (0.0–0.2 Hz), and behavioural whisking frequencies (1–20 Hz^43^).

**Figure 7:**
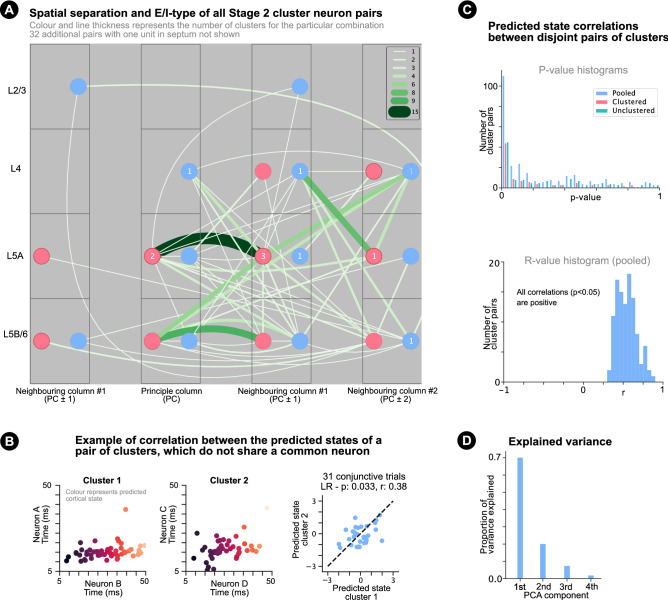
Neuron pair spatial separation and cortical state correlation. (**a**) Neuron pair spatial separation for criteria fulfilling correlated clusters (both stationary and ARIMA modelled). Line colour and thickness indicates the number of criteria fulfilling correlated clusters found for pairs of neurons in different layers and barrels and of different E/I-type. Red and blue correspond to E and I neuron types respectively. The numbers in the circles indicate the number of pairs of the same E/I type, layer and barrel. 32 neuron pairs were not added to the plot because at least one of the neurons in the pair lied in a septum (i.e. between barrels). (**b**) Example of two clusters which do not share a common neuron but have correlated cortical states over trials of a single stimulus. Left and centre: cluster spike times of two clusters coloured by predicted cortical state. Right: predicted cortical states for the two clusters are correlated on trials on which all 4 neurons spike. (**c**) Top: histograms showing *p*-values of linear correlation between predicted states of pairs of correlated pairs of neurons, which do not share a common neuron. Inclusion criteria: 15 conjunctive trials. Cases: clustered (redorange), unclustered (green) and clustered and unclustered pooled (blue). Bottom: r-value histograms of linear state correlations (clustered and unclustered pooled). (**d**) Mean variance explained by each PCA dimension between groups of 4 correlated neurons (on trials that all 4 neurons spiked).

Spike time correlations (stationary and ARIMA trend removed) were found for E–E, I–I and E–I neuron pairs separated over multiple (up to 4) cortical columns and layers (Fig. 7a, Supplementary Fig. 11-2), demonstrating shared non-adaptative trial-to-trial changes in the excitability of neuronal pairs over a large spatial distance. This introduces the possibility that spatially broad population-wide changes in neuronal excitability could modulate decoding neurons and allow them to respond in an state-dependent manner. The most correlations were found between E-type neurons in L5A and L5B/6 of the principal and neighbouring columns, although differences in the number of spike sorted neurons extracted by layer and E/I type must be considered (Supplementary Fig. 2). Low numbers of correlated clusters were found for neuron pairs within the same group, which could be a consequence of the local spike overlapping problem (i.e. spike detection difficulties when two or more neurons fire in close temporal and spatial proximity). For 32 neuron pairs, at least one neuron lied in a septum (i.e. between barrels).

### Correlation of predicted cortical state

Co-modulation of spike times for pairs of neurons separated over multiple cortical columns and layers introduced the possibility that spike time patterns are modulated by low-dimensional non-adaptative trial-to-trial population-wide changes in neuronal excitability. For the remaining analyses, Stage 2 clusters and correlated unclustered response distributions, which were stationary or made stationary, were pooled. If a cluster in the pool came from a correlated unclustered response distribution also in the pool, the correlated unclustered response distribution (rather than the cluster) was removed from the pool so that there was only one example per response distribution in the pool.

As an initial test, the value of each spike pair along the cluster’s (or unclustered response distribution’s) first principal component enabled a prediction of population-wide state (i.e. excitability) on a single trial (Fig. 7b). Such predicted single trial states were correlated over trials of single stimulus conditions for a large proportion of pairs of clusters and unclustered response distributions, which did not share a common neuron (Fig. 7c, top). Moreover, all statistically significant correlations $(p<0.05)$ were positive (Fig. 7c, bottom), providing evidence that trial-to-trial changes in excitability modulate the spike times of up to 4 neurons. Figure 7d also shows that the first PCA dimension explains $\sim 70\%$ of explainable spike time variance between groups of 4 neurons (on trials that all 4 neurons spiked). Together, these results strengthen the possibility that spike time patterns are modulated by low-dimensional non-adaptative trial-to-trial population-wide changes in neuronal excitability.

### Spike time and spike time difference prediction

**Figure 8:**
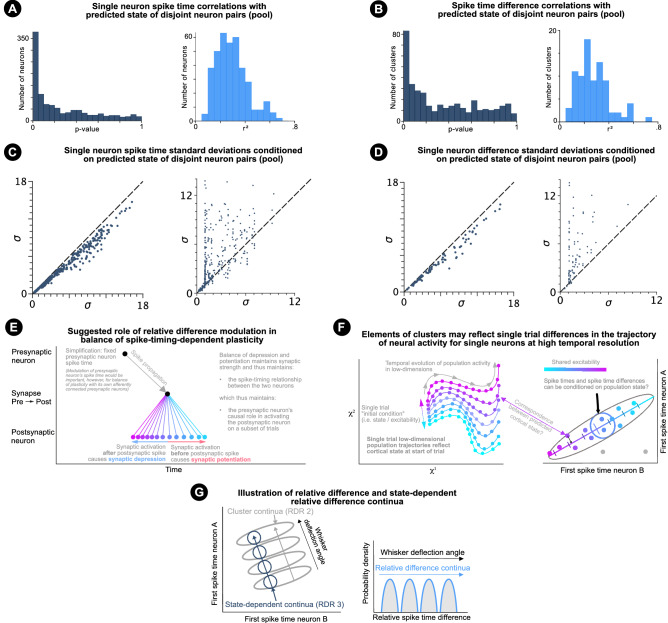
State-conditioned spike times and differences. Discussion. (**a**) Left: *p*-value histogram of correlations between predicted states of single correlated neuron pairs with the spike times of other correlated neuron pairs (clustered and unclustered pooled). Right: r-value histogram of statistically significant (*p* < 0.05) correlations. (**b**) Same as (**a**) but for correlations with spike time differences with non-$45^{\circ }$ correlation angles. (**c**) Left: variability comparison. X-axis shows the standard deviation of single neuron spike times within stationary and made-stationary clusters and correlated unclustered response distributions (pooled). Y-axis shows the standard deviations of spike times conditioned on the predicted state of single correlated neuron pairs, which do not share a common neuron. Right: Comparison of predicted state-dependent spike time variability predicted by factor analysis (x-axis) vs spike time variability conditioned on the predicted state of single correlated neuron pairs, which do not share a common neuron (y-axis). (**d**) Same as (**c**) but for correlations with spike time differences with non-$45^{\circ }$ correlation angles. (**e**) Suggested role of relative difference modulation in balancing spike-timing-dependent plasticity. A fixed spike time is shown for the presynaptic neuron, although the figure explains why the presynaptic neuron’s spike time should also be modulated. (**f**) Illustration of possible correspondence between single trial variability in low-dimensional population dynamics and single trial spike times for correlated clusters. (**g**) Left: Illustration of continua of first spike time distributions, corresponding to first spike times (not relative differences) under RDR 2 (grey) and 3 (dark blue). Right: Illustration of continua of relative spike time difference distributions, which could correspond to RDR 2 or 3.

Over trials of single stimulus conditions, the predicted state of single correlated neuron pairs is correlated with the spike times (Fig. 8a) and spike time differences (Fig. 8b) of other correlated neuron pairs, which do not share a common neuron. Figure 8c (left) demonstrates a reduction in variability when spike times are conditioned on the predicted state of other single correlated neuron pairs. In some cases, such state-conditioned spike time variability was on a similar order to that predicted by the factor analysis model above (Fig. 8c, right). A reduction in spike time difference variability is also seen when non-$45^{\circ }$ correlations are conditioned on the predicted state of other correlated single neuron pairs (Fig. 8d, left), although variability was mostly higher than the variability predicted by the factor analysis model under RDR 3. It is important to note that there is inherent noise in the state predicted from single pairs of neurons, which hampers spike time and spike time difference prediction. Future work may explore improved estimates of single trial state.

### Discussion

‘Time-warped multi-neuron single spike patterns’ describes the novel finding that, after clustering and control for adaptation, pairwise relative spike time differences are ‘warped’ by trial-to-trial changes in the excitability of neuron pairs (Figs. 4f, 6b) separated over multiple cortical layers and columns (Fig. 7a). The results show that clustering, non-$45^{\circ }$ noise correlations and adaptation, together, explain a large proportion of spike time variability and should be considered collectively when aiming to detect spike time relative difference representations. Correlations between the predicted states of pairs of clusters not sharing a common neuron (4 neurons total; Fig. 7c), strengthen the possibility that low-dimensional non-adaptative trial-to-trial population-wide changes in neuronal excitability modulate spike time patterns.

The study differs from revealment of oscillatory activity and spike train alignment to behavioural events through optimisation of population-wide shift and/or stretch functions^44^, firing rate time-warp modelling^45^ and spike train alignment to behavioural events^46^. Here we characterise the millisecond and sub-millisecond structure of spike-timing stimulus-response distributions, whilst accounting for clustering and non-stationarity. A novel form of sensory representation (time-warped multi-neuron single spike patterns) is proposed with a potential for sub-millisecond relative spike time difference encoding (Fig. 5). Non-$45^{\circ }$ correlation angles also demonstrate different time-warp functions for the spike times of different neurons. One previous study modelled time-warp-invariant integration^47^, which differs from state-dependent processing.

Sources of noise correlations are reviewed in^48^. Subthreshold membrane potential correlations are a prime candidate to underlie spike time noise correlations caused by trial-to-trial changes in shared excitability, as they are correlated across areas^49^ and affect action potential initiation^50^. Heterogeneous relationships between membrane potential changes and spike time latency could underlie non-$45^{\circ }$ correlations. The results open the opportunity to study the relationship between stimulus-evoked spike time noise correlations and noise correlations during spontaneous activity (previously characterised for the dataset^32^). We suggest that the purpose of relative spike time difference modulation, may be to maintain spike time patterns/relationships, by balancing spike-timing-dependent synaptic depression and potentiation (Fig. 8e).

Single trial variability in population dynamics is thought to reflect differences in population excitability or state at the start of a trial^27^. As such, population dynamics may offer a proxy for a cortical state, which modulates spike time patterns. Furthermore, the characterised spike timing variability may reflect the relationship between variability in population dynamics and single neuron spike time (and pairwise spike time difference) variability. In another sense, the characterised spike timing variability may reflect variability in the trajectory of population dynamics at a single neuron level and at high temporal resolution (Fig. 8f). Indeed, the characterisation of shared variability on a continuous spike timing scale could offer the opportunity to study population dynamics without loss of information through discretisation of neuronal responses, which typically uses bins of 10-20ms. The precise structure and latent factor dependence of relative difference representations uncovered here suggests that such representations may encode information that is additional to information decodable from classical binned spike count analysis (including classical modelling of population dynamics).

The results offer a large potential improvement for discriminable encoding under fixed-state-decoding and state-dependent-decoding regimes. Testing of decoding performance was limited by low cluster counts per stimulus condition and variability in cluster detection between stimulus conditions. For example, most clusters were detected at 0–4 Hz stimulations (Fig. 6e) and clusters were unlikely to be detected for many stimulus conditions for the same neuron pair, likely due to more non-spiking trials for higher stimulation frequencies due to spike count adaptation above 4 Hz^26,32,36,37^ (Fig. 2d). This does not imply the characterised form of representation is not utilised at higher frequencies; but means that fewer samples are available for characterisation at higher frequencies.

The detection of noise correlations over multiple cortical layers and columns (Fig. 7a) between groups of up to 4 neurons (Fig. 7c) suggests that trial-to-trial population-wide changes in neuronal excitability could be low-dimensional. This would increase the likelihood that downstream neurons were modulated by the same changes and could respond in a state-dependent manner. Determining the dimensionality of excitability changes requires characterisation of noise correlations over larger groups of neurons. Determining the dimensionality of single spike pattern noise correlations is made challenging by the observed clustered nature of pairwise spike time response distributions and low spiking reliability for single neurons (Fig. 2c). The observed clusters, however, offer the opportunity to to study a reliable part of stimulus representations, and to explore the dimensionality of spike time pattern modulation.

Future exploration of the characterised phenomena, including determining dimensionality of excitability changes and assessing encoding performance, would benefit from high trial counts and experimental paradigms designed to minimise adaptation^51^. Use of dynamic non-stationary and non-parametric clustering may also be beneficial both in the presence and absence of adaptation (Methods).

As the central goal of the manuscript was to extract isolated regions of high response probability, we chose to use DBSCAN as this is known to perform more robustly than GMMs at capturing the structure of the underlying probability density function when regions are non-Gaussian^39^. For example, because adaptation was likely to affect the shape of these regions by increasing latencies over trials, it was important to extract regions of high density for further analysis independently of an assumption of their structure. The non-Gaussianity of the isolated regions of high response probability was confirmed for a proportion of the extracted clusters. The first principal component and its angle offered a first approximation of the relationship of spike times and spike time differences with trial-to-trial changes in shared excitability (for the isolated regions of high response probability). Moreover, this approximation was able to explain the variance in spike times of other neuron pairs (Fig. 8a). Compared to the unclustered case, clustering better captured isolated regions of high-response probability for application of PCA, leading to novel detection of correlations and more precise cluster angle estimation (Fig. 4c). Moreover, a large proportion of clusters determined as Gaussian had correlation angles different from $45^{\circ }$ . Future work may aim to model non-linear relationships with underlying state, which in turn could enable more precise estimations of single trial state.

The encoding performance of RDR 2 and 3 would be best assessed over a range of similar stimuli. For example, small changes in whisker stimulation angle are likely to elicit slight changes to the response distributions, whilst not drastically changing the number of spiking trials per neuron. This would improve similarity in the number of clusters detected per stimulus and neuron pair. It can then be tested which response distribution properties improve decoding of transformation and correlate with behavioural performance. It is predicted that cluster (RDR 2) or state-dependent relative difference distributions (RDR 3) may change continuously with continuous stimulus transformations (Fig. 8g). More broadly, we propose that continua may be encoded in the cortex by time-warped relative spike time differences.

## Methods

### Neurophysiology

The dataset used for this paper is reused from earlier studies, of which analyses, data collection and spike sorting methods have previously been reported^5,12,32,52^.

In summary, extracellular recordings were performed in 18 anaesthetised adult Wistar rats using 16 or 128 electrode silicon probes (see below), inserted perpendicularly into the cortical representation of the whiskers in the primary somatosensory cortex, the so-called barrel cortex. Anaesthesia minimised the impact of motor signals and controlled for movement, which is a known source of variability in sensory cortices^53^. Neural latencies in the principal barrel column are unaffected by the level of anaesthesia^54^. Single whisker deflections were made at various frequencies (0.066-10Hz) in blocks, each containing typically 200 trials (sweeps) with an inter trial interval of 1/f. This was repeated for each of the selected target whiskers (maximum 4). Experimental sessions varied in terms of electrode placement, stimulated whiskers, stimulation frequencies and number of trials per unique stimulus. 12 experimental sessions stimulated at all frequency groups (0.066–0.2, 1, 2, ..., 10 Hz), 2 at low frequencies and a subset of higher frequencies (0.066–0.2, 1, 2, 4, 7 and 10 Hz), and 4 at low frequencies only (0.066–0.2 Hz).

Data from all experimental sessions were used in all analyses. 8 experimental sessions used single shanks with 16 electrodes, whilst 10 experimental sessions used 8 neighbouring shanks with 16 electrodes per shank covering 2–4 barrel-related columns (median 4). Neurons were classified as excitatory or inhibitory and assigned to a cortical layer L2/3, L4, L5A or L5B/6. Spiking activity is analysed following cortical activation onset, determined as 5.4 ms following stimulus onset (Supplementary Fig. 12).

Spikes were detected using groups of 2 to 4 channels defined as ‘virtual tetrodes’^55,56^. Spike detection was performed using amplitude-thresholding^57^. Spike sorting was performed leveraging waveform feature engineering and open source software^55,58^. Neuron quality was verified using several additional steps^57^. Cells were classified as putative excitatory (E) and inhibitory (I) neurons based on the mean spike waveform^59–61^. Voltage-sensitive dye (VSD) imaging, current-source density analyses and histology were used to identify the appropriate whisker-related cortical column and to determine the three-dimensional location of electrodes so that each neuron could be assigned to one cortical layer (L2/3, L4, L5A, L5B/6).

### Stage 1 clusters

*1. DBSCAN clustering.* A Python implementation^62^ of DBSCAN^38,39^ was used to detect clusters in first spike pair response distributions. Increasing the epsilon parameter increases the resulting cluster sizes. Clustering was performed using the following epsilon values sequentially: [0.4, 0.45, ..., 5.0]. The colour of points in Supplementary Fig. 13 represents the clusters to which points were assigned for epsilon = 1.35. If the previous lower epsilon value produced the same clustering, then the new clustering was discarded. Stage 1 clusters with less than 30 elements were also discarded, although these data points may be part of larger clusters extracted for higher epsilon values. Checks for similar or identical *individual* clusters were not made at this stage to avoid sampling bias, which could affect the uniformity of surrogate control tests.

*2. Isolation test.* This step aimed to ensure that clusters were not sub-parts of larger clusters but were instead well isolated clusters. For each cluster produced in step 1, an ‘intersection ellipse’ was created (dashed ellipses in Supplementary Fig. 13), with centre equal to the cluster mean and widths equal to $w_i = 3*max(0.5, \sigma _i)$ for neurons $i=0,1$, where $\sigma _i$ is the standard deviation of samples in the cluster for neuron i. Any cluster with an intersection ellipse within or intersecting with another intersection ellipse was excluded from further analysis. In Supplementary Fig. 13, this corresponded to the blue, red and light blue dashed clusters.

*3. Stage 1 clusters.* For each remaining cluster, a flat bounding ellipse was created (solid green ellipse in Supplementary Fig. 13) with centre equal to the cluster mean, and widths equal to $w_i = 4*\sigma _i$ for neurons $i=0,1$, where $\sigma _i$ is the standard deviation of samples in the cluster for neuron i. All elements within the bounding ellipse were assigned to the Stage 1 cluster. The use of non-angled ellipses was necessary for a controlled test of cluster correlations.

As 94.6% of Stage 2 clusters extracted over 90ms following cortical activation onset lay within 50ms following onset (Supplementary Fig. 12), analysis was constrained to spikes in the first 50ms following activation onset.

### Correlation surrogate control datasets

To test that correlations were not artifacts of the clustering algorithm and were above chance levels, the algorithm was applied to the following surrogate control datasets in which correlated clusters appeared at chance levels (Supplementary Fig. 5-1):

*(i) All shuffled.* Pairwise shuffling of elements of first spike pair distribution. For each first spike pair stimulus-response distribution, first spike pairs are shuffled so that each spike time of one neuron is matched with a different spike time of the other neuron.

*(ii) Single Gaussian.* Spike pairs drawn from an independent bivariate normal distribution with the same mean and standard deviation as the original spikes. The number of samples drawn from the distribution was equal to the number of original samples.

### Stage 2 clusters

*4. Angled ellipses.* An angled ‘Stage 2’ ellipse was calculated to better estimate the distribution underlying each correlated $(p<0.005)$ Stage 1 cluster using a 3-stage bootstrap procedure. In the bootstrap step 1, a number of samples were taken with replacement from the Stage 1 cluster equal to the number of samples in the Stage 1 cluster. PCA was performed on the sample to attain a sample mean and covariance matrix. 10,000 such bootstrap iterations were made. The sample means and covariance matrices were then averaged to construct a new bounding ellipse (similar to the rotated green ellipse in Supplementary Fig. 13) with centre equal to the bootstrapped mean, rotation equal to the angle that the principal component made with the positive x-axis, and widths equal to $4\sigma _i$ where $\sigma _i$ is the square root of the variance explained by each principal component.

The bootstrap procedure was then repeated by sampling the elements within this new ellipse (bootstrap step 2), with the averaged mean and covariance matrix used to construct a third ellipse for sampling in bootstrap step 3. Elements within the final bounding ellipse defined by the averaged mean and covariance matrix of bootstrap step 3 were assigned to the Stage 2 cluster.

*5. Cluster similarity check.* Angled clusters which shared at least 15 elements to previous angled clusters (produced by lower epsilon values for the same response distribution) were excluded from further analysis. This means that no clusters were included, which contained well isolated ‘sub-clusters’ produced by lower epsilon values. As a result, all extracted Stage 2 clusters were from unique response distributions, guaranteeing that there were no repeated clusters.

### Correlation angle

The 10,000 sample first principal components produced in bootstrap stage 3 were used to estimate the angle that the first principal component makes with the positive x-axis (with empirical confidence intervals). To account for symmetry, sample principal components with negative y-components were replaced by their anti-correlated vector so that all vectors had angles with the positive x-axis between 0 and $180^{\circ }$. To allow averaging and angular difference calculations without the effects of discontinuity, the vectors were rotated anti-clockwise about the origin such that the anticlockwise angles of the vectors with the positive x-axis were between 0 and $360^{\circ }$ (double their original angle). A mean first principal component vector was then calculated in this space by averaging over the bootstrap samples. For each cluster, the 250th and 9750th largest bootstrap sample angles were used to define a 95% empirical confidence interval (in this $360^{\circ }$ space). The resulting mean vector was then rotated clockwise by half of its anti-clockwise angle with the positive x-axis so that it was between 0 and $180^{\circ }$. This was used as the final $\theta$ angle for the cluster. The confidence interval bounds were divided by 2 to create angle confidence intervals for the first principal component on the correct scale.

### Cluster angle surrogate control datasets

The following two angle surrogate control datasets were created to verify the main clustering algorithm and compare it to the unclustered case:

*(i)* $45^{\circ }$ *control.* To validate that differences from $45^{\circ }$ were not artifacts of the algorithm, a surrogate control dataset was created by replacing each Stage 2 cluster and all samples within a $6\sigma$ circle of its mean (where $\sigma$ was equal to the mean of the cluster spike time standard deviations of the two neurons) with samples drawn from a $45^{\circ }$ 2D Gaussian. The algorithm found correlated clusters with angles different from $45^{\circ }$ at chance-levels (Supplementary Fig. 6).

*(ii) 2nd distractor cluster.* An additional surrogate control dataset was created to demonstrate that the clustering algorithm, in the presence of a second uncorrelated cluster, detects the first correlated cluster and calculates its correct correlation angle (Supplementary Fig. 6-2). For each response distribution in which a Stage 2 cluster was extracted by the main clustering algorithm, a new response distribution was created with two new clusters. The first cluster was created by sampling a number of samples equal to the number of samples in the original Stage 2 cluster, drawn from a $45^{\circ }$ Gaussian centred at the mean of the original Stage 2 cluster, with the new cluster spike time standard deviations of the two neurons equal to the mean of their original standard deviations. The second cluster was created by sampling a number of samples equal to the number of samples in the original Stage 2 cluster, drawn from an uncorrelated 2D Gaussian centred to the right at a $15^{\circ }$ angle from the first cluster with the cluster spike time standard deviations of the two neurons equal to those of the original Stage 2 cluster. The x coordinate of the mean point was equal to the mean of the original Stage 2 cluster plus 10 standard deviations of the x-axis neuron’s cluster spike times. For this surrogate control dataset, the clustering algorithm detected clusters with correlation angles different from $45^{\circ }$ at chance-levels (Supplementary Fig. 6-2).

This differed to the unclustered approach, which found correlations with angles $\sim 15^{\circ }$, highlighting an advantage of the clustering algorithm over the unclustered approach.

The Henze–Zirkler null hypothesis of normality was rejected $(p < 0.05)$ for 6.5% (11/169) and 7.2% (12/166) of the Gaussian sampled clusters detected in the two angle control datasets, verifying that the algorithm and Henze–Zirkler test could detect normally distributed clusters.

### Comparison to alternative clustering methods

The clustering algorithm was compared to the following two Gaussian Mixture Model approaches:

*(i) Standard GMM.* The first approach uses the classical Bayesian information criterion (BIC) to select an optimal number of Gaussians (between 1 and 10) for the mixture model of each pairwise response distribution. This approach was therefore not constrained to find isolated regions of high response probability. Indeed, for the initial Stage 1 clustering test, this algorithm detected clusters, which were fragmentations of regions of high-response probability (e.g. Supplementary Fig. 5-2), rather than isolated regions of high response probability (which were the focus of the study). In fact, the algorithm was biased to detect correlations, such that it found above-chance correlations for the two correlation control datasets (Supplementary Fig. 5-2). This made it unsuitable for detecting correlated clusters in the main dataset.

*(ii) Custom GMM.* An augmented GMM approach was used in which steps 2-4 of the main clustering algorithm were additionally applied after the initial BIC based GMM clustering step (i). This clustering type therefore included checks for isolation. Supplementary Fig. 5-3a shows that correlations were found at chance-levels after the addition of these steps. However, almost all of the detected clusters included all samples from their originating response distribution (Supplementary Fig. 5-3b), showing that the algorithm did not find isolated regions of high-response probability that contained less than 95% of the samples in the original sample distribution. The algorithm did, however, find clusters with correlation angles different from $45^{\circ }$ both before and after checks for stationarity (Supplementary Fig. 5-3c), although considerably fewer than the main clustering algorithm and unclustered approach. The Henze–Zirkler null hypothesis of normality was rejected $(p < 0.05)$ for only 31.9% (15/47) of Stage 2 clusters extracted by the Custom GMM algorithm, confirming that the Custom GMM algorithm was biased towards detecting clusters likely to be Gaussian distributed. The algorithm was also verified for the two angle control datasets (Supplementary Fig. 6-3). Moreover, the Henze–Zirkler null hypothesis of normality was rejected $(p < 0.05)$ for 3.8% (1/26) and 3.3% (1/30) of the Gaussian sampled clusters detected in the two angle control datasets, further verifying the Henze–Zirkler test of normality when used in combination with clustering.

### Stationary cluster and factor analysis criteria

Clusters were determined to be stationary and sufficiently correlated such that trial-to-trial changes in shared excitability could underlie the covariation if they passed the following tests:

1. Cluster spike times were correlated: linear regression *p*-value < 0.005, r-value > 0.3.
2. Single neuron cluster spike times were not linearly correlated with trial index (*p*-value > 0.05).
3. The cluster correlation was not explained by trial index. Particularly, a linear regressor predicting spike pairs from trial index must have $r^2 < 0.05$.
4. Both neurons KPSS stationary: *p*-value > 0.05. The Kwiatkowski–Phillips–Schmidt–Shin trend stationarity test (KPSS) tested whether cluster first spike times of single neurons were stationary around a deterministic trend.
5. Cluster spike times of both neurons have a trial-lag-1 autocorrelation *p*-value > 0.05 (this requirement was relaxed for the differenced clusters but not ARIMA clusters). The trial-lag-1 autocorrelation describes the following. Consider the cluster spike times $s_{j,t}$ of neurons j = 0,1 on cluster trials t = 1,...,T where T is the number of cluster trials for the cluster. Trial-lag-1 autocorrelations tests the correlations between single neuron cluster spike times on trials t with spike times on trial t+1.
6. Bartlett sphericity *p*-value > 0.05. Bartlett sphericity tests that the correlation matrix is significantly different from the identity, and is a standard pre-test for factor analysis.
7. The factor correlation matrix had only one eigenvalue greater than 1, suggesting that a single factor underlies each cluster correlation.

### Factor analysis angle and difference distribution

$\theta$ and $\theta _{45}$ angles of factor analysis mean lines were estimated using the same technique as for PCA angles. Factor analysis $\theta$ angles were similar to the PCA $\theta$ angles for stationary, differenced and ARIMA modelled clusters (Supplementary Fig. 11).

As the factor analysis model assumes that noise for each neuron given $\eta$ is normally distributed and independent between neurons, the state-dependent spike time relative difference standard deviation predicted by the model is equal to $\sigma _{diff} = \sqrt{\sigma _{0}^2 + \sigma _{1}^2}$ where $\sigma _i$ is the state-dependent standard deviation of neuron *i* predicted by factor analysis.

### Differencing

Differencing was applied to single neuron cluster spike times if any of the following stationarity criteria were not met for the cluster spike times of either neuron: (1) Trial index vs spike time linear regression *p*-value > 0.05, (2) KPSS stationarity *p*-value > 0.05, (3) Trial-lag-1 autocorrelation *p*-value > 0.05.

Differencing a series of first spike times subtracts the first spike time on trial t-1 from the first spike time trial t. Differencing reduces the number of samples by 1. For clusters for which the spike times of only one neuron was differenced, the first sample of the undifferenced neuron was removed, so that the number of samples was equal for both neurons. In this case a spike time for an undifferenced trial t was matched with the difference between the spike times on trial t and trial t−1.

Differencing increases the standard deviation of a time series $S = (s_1, \ldots , s_t)$ by approximately $\sqrt{2}$. This is shown by considering differencing as approximate to negating two independent random variables $S_{(1, t-1)} = (s_1, \ldots , s_{t-1})$ and $S_{(2, t)} = (s_2, \ldots , s_t)$, where *t* is the number of samples in the original time series. As the standard deviation of $S_{(0, t-1)}$ and $S_{(1, t)}$ are approximate to the standard deviation of the original series *S*, the standard deviation of the differences is on the order of $\sigma _{differenced} \approx \sqrt{\sigma _{(1, t-1)}^2 + \sigma _{(2, t)}^2} \approx \sqrt{2\sigma ^2} = \sqrt{2}\sigma$ where $\sigma$ is the standard deviation of the original time series. Differenced time series were therefore divided by $\sqrt{2}$ to return the differenced time series to the scale of the original time series, which comparison to ARIMA models shows to be reasonable (Fig. 4f). The assessment of ‘raw’ differences, rather than the ARIMA residuals, however, allows a direct assessment of cluster angles after trend removal.

Differencing was applied to the spike times of clusters rather than to all first spike times of a single neuron in response to a single stimulus. This was to ensure that differences were calculated between spike times of the same trials for both neurons in a pair. That is, the difference between spike times on trial X and spike times on trial Y were compared for neuron pairs. Differencing was not alternatively applied to all samples of a first spike stimulus-response distribution, as this could give rise to correlations caused by switching between clusters.

### Automated Box-Jenkins method

To determine the order of autoregressive (AR) and moving average (MA) components, the Box-Jenkins method is classically applied manually based on observation of autocorrelations and partial autocorrelations. To deal with the negative autocorrelations introduced by differencing and to account for other autocorrelations, an automated and simplified version was developed based on the description of the Box-Jenkins method found at^63^.

For determination of the MA order, if the trial-lag-1 autocorrelation is negative and significant, the series is slightly overdifferenced and the Box-Jenkins method checks for a sudden cut off in the autocorrelation plot. The lag at which there is a cut-off is used as the MA order. To automate this, the algorithm checked if the trial-lag-1 autocorrelation was negative ($r < -0.1$). If so, the *p*-values of lagged autocorrelations were tested (lags 1–5). If there was a sudden increase in *p*-value from below 0.05, from one lag to another, by more than 0.15, then the value of the last significant autocorrelation was used as the MA order.

For determination of the AR order, if the trial-lag-1 autocorrelation is positive ($r > 0.1$) and significant, the series is slightly underdifferenced, and the Box-Jenkins method checks similarly for a sudden cut-off in the partial-autocorrelation plot to determine the AR term.

Figure 5c confirms that the majority of ARIMA applications were of simple low-order MA components, as expected for the differenced time series. Moreover, autocorrelations and cross-correlations were reduced to chance-levels by ARIMA application, and ARIMA estimated cluster angles were similar to the original clusters (Supplementary Fig. 11).

### Further discussion of methods

To control the possible ill-posedness and the robustness problem of the underlying clustering problem in non-Gaussian situations with strong noise and small data samples, a robust clustering pipeline was designed which took into account the level of noise present in the data and the low number of samples in each response distribution. It follows that surrogate control datasets should match the general statistics of the response distributions. For these reasons surrogate control datasets were created by transforming pre-existing response distributions.

The joint roles of adaptation and trial-to-trial changes in excitability promote the use of dynamic clustering techniques in future work where adaptation is present^64^. The multiscale character of the underlying neural processes introduces the possibility of low-dimensional temporally persistent latent regime-switching processes, which are a common source of non-stationarity across nature^64^ and are also commonly ignored by analysis techniques^65^. Such processes are likely to contribute to the complexity of the observed time series, including clusters.

Techniques, which address these issues for limited sample sizes (i.e.^66^) should be applied in future research where adaptation is present to (i) characterise the multi-scale nature of regime switching processes, (ii) estimate stimulus representation independently of adaptation and invisible biases caused by multi-scale processes beyond depressive spike time latency adaptation^65^ and (iii) characterise the dimensionality of trial-to-trial changes in excitability . Understanding of (i) and (ii) will allow stricter control of adaptative and multi-scale processes for the understanding of (iii). This paper aimed to take a first step towards understanding such dynamical processes by characterising a reliable subset of responses (clusters).

## Supplementary Information

Supplementary Information.

Supplementary Video 1

Supplementary Video 2

## Data Availability

All code and data needed to reproduce all figures of the manuscript are available at https://github.com/james-isbister/spikewarp.git and https://github.com/james-isbister/IsbisterEtAl-SciRep2021-Data.git.

## References

[1] Gerstner W, Kreiter AK, Markram H, Herz AV (1997). Neural codes: Firing rates and beyond. Proc. Natl. Acad. Sci..

[2] Johnson KO (2000). Neural coding. Neuron.

[3] Gerstner W, Kistler WM, Naud R, Paninski L (2014). Neuronal Dynamics: From Single Neurons to Networks and Models of Cognition.

[4] Brette R (2015). Philosophy of the spike: Rate-based vs. spike-based theories of the brain. Front. Syst. Neurosci..

[5] Reyes-Puerta V (2015). Long-range intralaminar noise correlations in the barrel cortex. J. Neurophys..

[6] Zuo Y (2015). Complementary contributions of spike timing and spike rate to perceptual decisions in rat S1 and S2 cortex. Current Biol..

[7] Uzzell V, Chichilnisky E (2004). Precision of spike trains in primate retinal ganglion cells. J. Neurophysiol..

[8] Johansson RS, Birznieks I (2004). First spikes in ensembles of human tactile afferents code complex spatial fingertip events. Nat. Neurosci..

[9] Gollisch T, Meister M (2008). Rapid neural coding in the retina with relative spike latencies. Science.

[10] Storchi, R., Bale, M., Biella, G. & Petersen, R. Comparison of latency and rate coding for the direction of whisker deflection in the subcortical somatosensory pathway. *J. neurophysiol.* **108**(7), 1810–21 (2012). 10.1152/jn.00921.2011 PMC3545005 22815402

[11] Reinagel P, Reid RC (2002). Precise firing events are conserved across neurons. J. Neurosci..

[12] Reyes-Puerta V, Sun J-J, Kim S, Kilb W, Luhmann HJ (2014). Laminar and columnar structure of sensory-evoked multineuronal spike sequences in adult rat barrel cortex in vivo. Cerebral Cortex.

[13] Panzeri S, Petersen RS, Schultz SR, Lebedev M, Diamond ME (2001). The role of spike timing in the coding of stimulus location in rat somatosensory cortex. Neuron.

[14] Shadlen MN, Newsome WT (1998). The variable discharge of cortical neurons: Implications for connectivity, computation, and information coding. J. Neurosci..

[15] Lin I-C, Okun M, Carandini M, Harris KD (2015). The nature of shared cortical variability. Neuron.

[16] Hong H, Yamins DL, Majaj NJ, DiCarlo JJ (2016). Explicit information for category-orthogonal object properties increases along the ventral stream. Nat. Neurosci..

[17] Chang L, Tsao DY (2017). The code for facial identity in the primate brain. Cell.

[18] Stringer, C., Michaelos, M., Tsyboulski, D., Lindo, S. E., & Pachitariu, M. High-precision coding in visual cortex. *Cell*, **184**(10), 2767–2778 (2021). 10.1016/j.cell.2021.03.042 33857423

[19] Gasparini S, Magee JC (2006). State-dependent dendritic computation in hippocampal CA1 pyramidal neurons. J. Neurosci..

[20] Branco T, Clark BA, Häusser M (2010). Dendritic discrimination of temporal input sequences in cortical neurons. Science.

[21] Branco T, Häusser M (2011). Synaptic integration gradients in single cortical pyramidal cell dendrites. Neuron.

[22] Markram H, Lübke J, Frotscher M, Sakmann B (1997). Regulation of synaptic efficacy by coincidence of postsy502 naptic APs and EPSPs. Science.

[23] Mainen ZF, Sejnowski TJ (1995). Reliability of spike timing in neocortical neurons. Science.

[24] Carandini M (2004). Amplification of trial-to-trial response variability by neurons in visual cortex. PLoS Biol..

[25] Branco T, Staras K (2009). The probability of neurotransmitter release: variability and feedback control at single synapses. Nat. Rev. Neurosci..

[26] Chung S, Li X, Nelson SB (2002). Short-term depression at thalamocortical synapses contributes to rapid adaptation of cortical sensory responses in vivo. Neuron.

[27] Kao JC (2015). Single-trial dynamics of motor cortex and their applications to brain-machine interfaces. Nat. Commun..

[28] Pandarinath C (2018). Inferring single-trial neural population dynamics using sequential auto-encoders. Nat. Methods.

[29] Arieli A, Sterkin A, Grinvald A, Aertsen A (1996). Dynamics of ongoing activity: Explanation of the large variability in evoked cortical responses. Science.

[30] Schölvinck ML, Saleem AB, Benucci A, Harris KD, Carandini M (2015). Cortical state determines global variability and correlations in visual cortex. J. Neurosci..

[31] Resulaj A, Ruediger S, Olsen SR, Scanziani M (2018). First spikes in visual cortex enable perceptual discrimina tion. Elife.

[32] Reyes-Puerta V (2015). High stimulus-related information in barrel cortex inhibitory interneurons. PLoS Comput. Biol..

[33] Feldmeyer D (2013). Barrel cortex function. Progr. Neurobiol..

[34] Abeles M (2012). Local Cortical Circuits: An Electrophysiological Study.

[35] Izhikevich EM (2006). Polychronization: Computation with spikes. Neural Comput..

[36] Khatri V, Hartings JA, Simons DJ (2004). Adaptation in thalamic barreloid and cortical barrel neurons to periodic whisker deflections varying in frequency and velocity. J. Neurophysiol..

[37] Wang Q, Webber RM, Stanley GB (2010). Thalamic synchrony and the adaptive gating of information flow to cortex. Nat. Neurosci..

[38] Ester M (1996). A density-based algorithm for discovering clusters in large spatial databases with noise. KDD.

[39] Rodriguez A, Laio A (2014). Clustering by fast search and find of density peaks. Science.

[40] Nau, R. Identifying the order of differencing in an ARIMA model. Retrieved July 13, 2021, from https://people.duke.edu/~rnau/411arim2.htm (2020).

[41] Ho S, Xie M (1998). The use of ARIMA models for reliability forecasting and analysis. Comput. Ind. Eng..

[42] Box GE, Jenkins GM, Reinsel GC, Ljung GM (2015). Time Series Analysis: Forecasting and Control.

[43] Carvell GE, Simons DJ (1990). Biometric analyses of vibrissal tactile discrimination in the rat. J. Neurosci..

[44] Williams AH (2020). Discovering precise temporal patterns in large-scale neural recordings through robust and interpretable time warping. Neuron.

[45] Lawlor PN, Perich MG, Miller LE, Kording KP (2018). Linear-nonlinear-time-warp-poisson models of neural activity. J. Comput. Neurosci..

[46] Shusterman, R., Sirotin, Y. B., Smear, M. C., Ahmadian, Y., & Rinberg, D. Sniff invariant odor coding. *Eneuro*, *5*(6). (2018). 10.1523/ENEURO.0149-18.2018 PMC6325545 30627641

[47] Gütig R, Sompolinsky H (2009). Time-warp-invariant neuronal processing. PLoS Biol..

[48] Doiron B, Litwin-Kumar A, Rosenbaum R, Ocker GK, Josic K (2016). The mechanics of state-dependent neural correlations. Nat. Neurosci..

[49] Poulet JF, Petersen CC (2008). Internal brain state regulates membrane potential synchrony in barrel cortex of behaving mice. Nature.

[50] Petersen CC, Hahn TT, Mehta M, Grinvald A, Sakmann B (2003). Interaction of sensory responses with spontaneous depolarization in layer 2/3 barrel cortex. Proc. Natl. Acad. Sci..

[51] Harris, K. D. Nonsense correlations in neuroscience. *bioRxiv*, 2020-11. (2021).

[52] Reyes-Puerta, V. et al. Propagation of spontaneous slow-wave activity across columns and layers of the adult rat barrel cortex in vivo. *Brain Struct. Funct.* **221**, 4429–4449 (2016). 10.1007/s00429-015-1173-x 26754838

[53] Stringer C (2019). Spontaneous behaviors drive multidimensional, brainwide activity. Science.

[54] Simons DJ, Carvell GE, Hershey AE, Bryant DP (1992). Responses of barrel cortex neurons in awake rats and effects of urethane anesthesia. Exp. Brain Res..

[55] Harris KD, Henze DA, Csicsvari J, Hirase H, Buzsaki G (2000). Accuracy of tetrode spike separation as determined by simultaneous intracellular and extracellular measurements. J. Neurophysiol..

[56] Einevoll GT, Franke F, Hagen E, Pouzat C, Harris KD (2012). Towards reliable spike-train recordings from thousands of neurons with multielectrodes. Current Opin. Neurobiol..

[57] Gray CM, Maldonado PE, Wilson M, McNaughton B (1995). Tetrodes markedly improve the reliability and yield of multiple single-unit isolation from multi-unit recordings in cat striate cortex. J. Neurosci. Methods.

[58] Hazan L, Zugaro M, Buzsáki G (2006). Klusters, NeuroScope, NDManager: a free software suite for neurophysio logical data processing and visualization. J. Neurosci. Methods.

[59] Sirota A (2008). Entrainment of neocortical neurons and gamma oscillations by the hippocampal theta rhythm. Neuron.

[60] Sakata S, Harris KD (2009). Laminar structure of spontaneous and sensory-evoked population activity in auditory cortex. Neuron.

[61] Royer S (2012). Control of timing, rate and bursts of hippocampal place cells by dendritic and somatic inhibition. Nat. Neurosci..

[62] Pedregosa F (2011). Scikit-learn: Machine Learning in Python. J. Mach. Learn. Res..

[63] Nau, R. Identifying the orders of AR and MA terms in an ARIMA model https://people.duke.edu/~rnau/ 411arim3.htm. Accessed 28 Oct 2020. (2020).

[64] Metzner P, Putzig L, Horenko I (2012). Analysis of persistent nonstationary time series and applications. Commun. Appl. Math. Comput. Sci..

[65] DeWiljes J, Putzig L, Horenko I (2014). Discrete nonhomogeneous and nonstationary logistic andMarkov regression models for spatiotemporal data with unresolved external influences. Commun. Appl. Math. Comput. Sci..

[66] Horenko I (2020). On a scalable entropic breaching of the overfitting barrier for small data problems in machine learning. Neural Comput..

