# Supplementary material for: Clustering and control for adaptation uncovers time-warped spike time patterns in cortical networks in vivo
Source: Sci Rep. 2021 Jul 29;11:15066. doi: 10.1038/s41598-021-94002-0 (PMC8322153; doi:10.1038/s41598-021-94002-0)
Supplement: Supplementary file 1 — Supplementary Information. [file 41598_2021_94002_MOESM1_ESM.pdf]

Title: Clustering and control for adaptation uncovers time-warped spike time patterns in cortical networks in vivo

Authors:

- James B. Isbister (* corresponding author)
  Department of Experimental Psychology, University of Oxford, Oxford, UK
  The Blue Brain Project, École Polytechnique Fédérale de Lausanne, 1202 Geneva, Switzerland
  james.isbister@epfl.ch, https://orcid.org/0000-0002-1013-3013

- Vicente Reyes-Puerta
  Institute of Physiology, University Medical Center, Johannes Gutenberg University, Mainz, Germany
  vr.research@outlook.com, https://orcid.org/0000-0001-9193-4308

- Jyh-Jang Sun
  NERF, Kapeldreef 75, 3001 Leuven, Belgium
  imec, Remisebosweg 1, 3001 Leuven, Belgium (current address)
  Institute of Physiology, University Medical Center, Johannes Gutenberg University, Mainz, Germany
  Jyh-Jang.Sun@nerf.be, https://orcid.org/0000-0002-2326-4864

- **Illia Horenko**
  Faculty of Informatics, Universita della Svizzera Italiana, Via G. Buffi 13, 6900 Lugano Switzerland
  illia.horenko@usi.ch, http://orcid.org/0000-0002-4964-8014

- **Heiko J. Luhmann**
  Institute of Physiology, University Medical Center, Johannes Gutenberg University, Mainz, Germany
  luhmann@uni-mainz.de, https://orcid.org/0000-0002-7934-8661

Abstract: How information in the nervous system is encoded by patterns of action potentials (i.e. spikes) remains an open question. Multi-neuron patterns of single spikes are a prime candidate for spike time encoding but their temporal variability requires further characterisation. Here we show how known sources of spike count variability affect spike time patterns between neurons separated over multiple layers and columns of adult rat somatosensory cortex in vivo. On subsets of trials (clusters) and after controlling for neuronal stimulus-response adaptation, spike time differences between pairs of neurons are 'time-warped' (compressed/stretched) by trial-to-trial changes in shared excitability, explaining why fixed spike time patterns and noise correlations are seldom reported. We show that the predicted cortical state is correlated between groups of 4 neurons, introducing the possibility of spike time pattern modulation by trial-to-trial changes in population-wide excitability (i.e. cortical state). Under the assumption of state-dependent decoding\color{black}\st{Under this assumption}, we propose an improved potential encoding capacity.

Keywords: Neural coding, precise spike timing, time-warp, state-dependent processing, barrel cortex, noise correlations, neural adaptation, in vivo recording

Supplementary Video 1 & 2 Legend. First spike response distributions and results of time series analysis for all Stage 2 clusters (Supplementary Video 1) and correlated unclustered response distributions (Supplementary Video 2). Description refers to Stage 2 clusters but same applies to correlated unclustered response distributions (but without clustering steps).

Top left: Sampled first spike response distribution for two neurons (for all pairs which produced a Stage 2 cluster). Point colours illustrate DBSCAN clusters. Black points are outliers. Solid flat ellipses illustrates 4σ boundaries of Stage 1 clusters. Angled ellipses illustrate 4σ boundary of Stage 2 clusters. Yellow ellipse illustrates 4σ ellipses under the assumption of independence and normality. For criteria fulfilling Stage 2 clusters, purple circle illustrates the 4σ boundary of the factor-conditioned response distribution predicted by factor analysis for a single factor value (i.e. 0). For criteria fulfilling Stage 2 clusters, the purple line shows the mean points predicted for the factor values [-4, 4] (i.e. with variance equal to 0).

Right: This subfigure analyses the spike times of the corresponding Stage 2 clusters. Columns 2 and 3 appear only for differenced Stage 2 clusters. Rows 1 and 2 corresponds with the example in Fig. 6a (see for further details). The columns correspond to (1) the original Stage 2 cluster spike times, (2) the cluster after non-stationary neuron cluster spike times were differenced, and (3) the result of applying AR and/or MA models to the differenced cluster. Row 1 shows the cluster spike times of both neurons. Row 2 shows the corresponding cluster, with the point colour illustrating the trial index (from dark to light).

If the clusters fulfil the criteria after differencing and/or ARIMA modelling, the purple 4σ factor analysis ellipse and line are shown in the corresponding row 2 plot. Rows 3 and 4 present the autocorrelation and partial autocorrelation plots respectively with 95% confidence intervals. Row 5 shows the QQ-plots for each neuron time series.

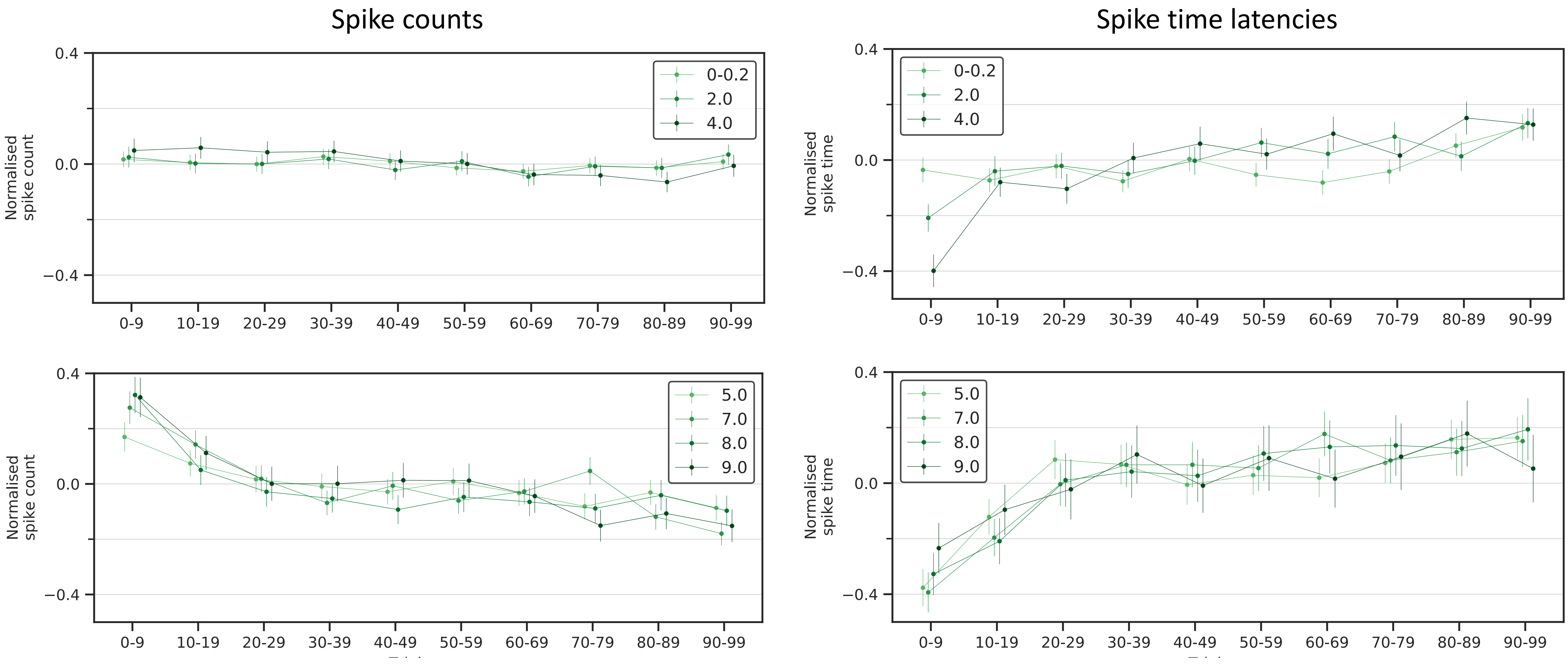

Supplementary Fig. 1. Spike count and spike time latency adaptation by remaining stimulation frequencies. As in Fig. 2d.

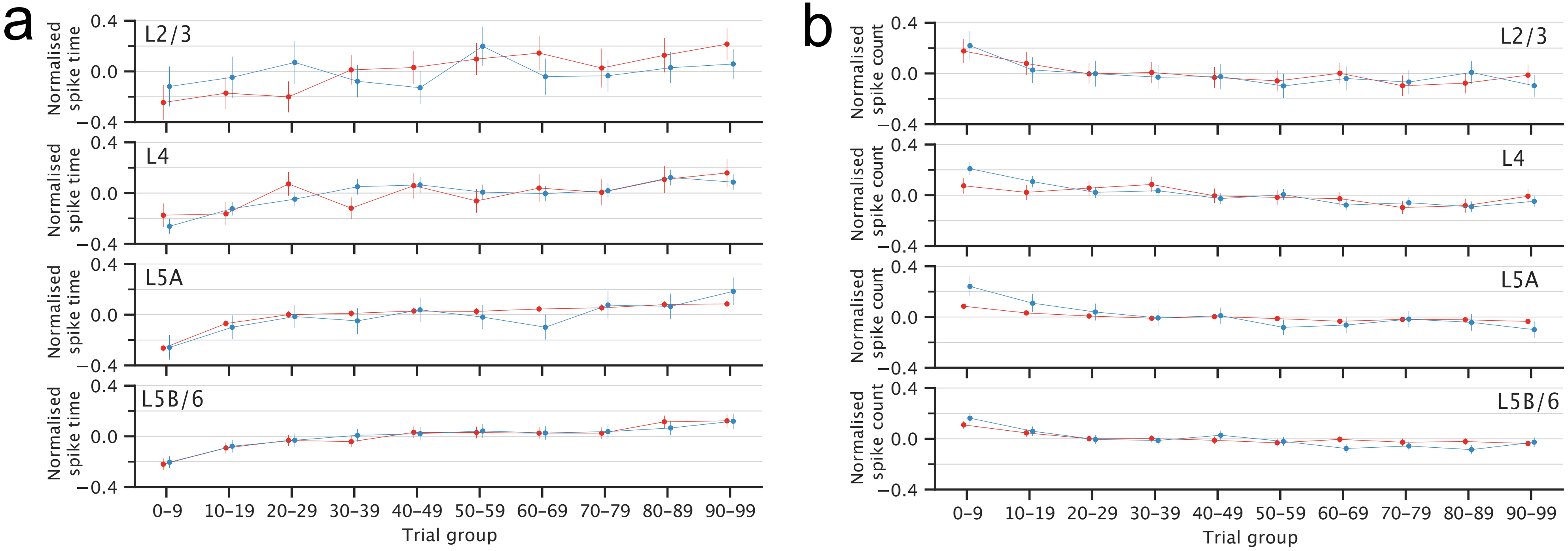

c

Number of spike sorted neurons by cortical layer and E/I type

	L2/3	L4	L5A	L5B
E	31	58	198	135
I	4	19	16	33

Supplementary Fig. 2. Spike time latency and spike count adaptation by neuron group.
(a) For each single neuron and stimulus condition combination, first spike times are normalised (combination mean subtracted followed by division by combination standard deviation). Means of these normalised first spike times are calculated for neuron groups and 10 trial bins (i.e. trials 0-9, ..., 90-99). These mean values are plotted with a 95\% confidence interval. Values for excitatory and inhibitory neuron groups shown in red and blue respectively. Note that L2/3 is undersampled due to sparse activity and responsiveness of L2/3 neurons (e.g. de Kock and Sakmann, PNAS, 2009). (b) Same as (a) but for normalised spike counts. (c) Table displaying the number of spike sorted neurons recorded over all experimental sessions by layer and E/I type.

de Kock, C. P., & Sakmann, B. (2009). Spiking in primary somatosensory cortex during natural whisking in awake head-restrained rats is cell-type specific. *Proceedings of the National Academy of Sciences*, *106*(38), 16446-16450.

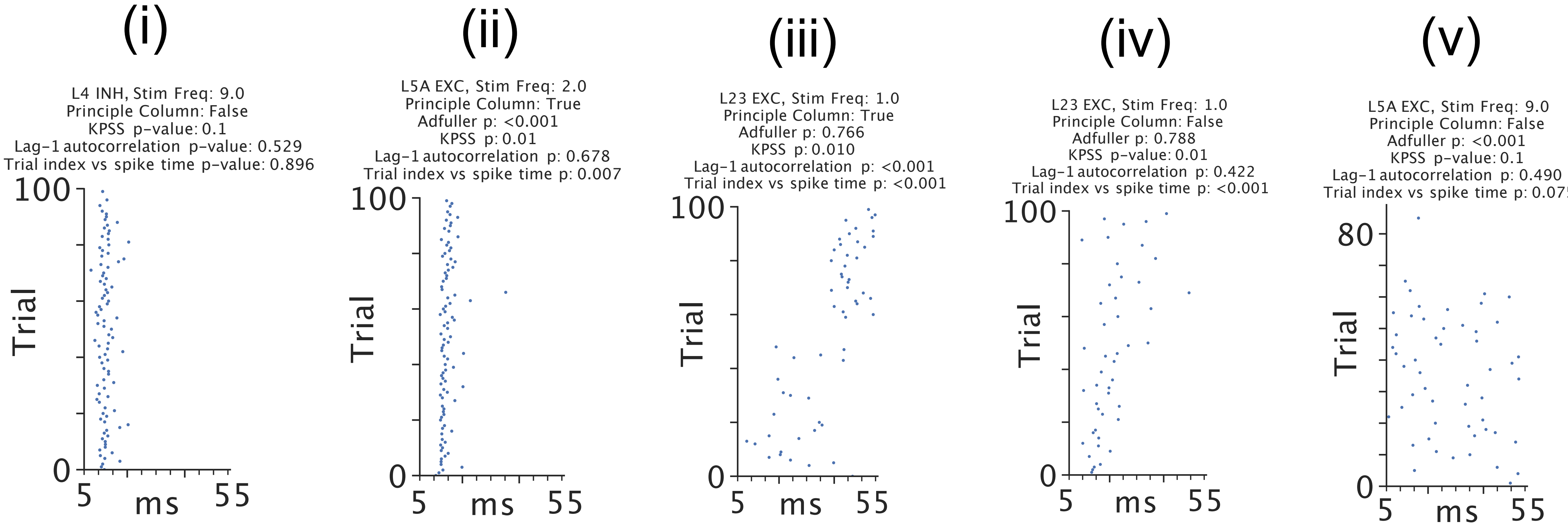

Supplementary Fig. 3 Heterogeneous first spike rasters following cortical onset for five single-neuron single-stimulus combinations. L4 INH (i) and L5A EXC neuron (ii) demonstrate reliable short-latency response between 5 and 10 ms poststimulus with prominent adaptation. L2/3 EXC neurons (iii and iv) also demonstrate adaptation but responses are more variable. In contrast, L5A EXC neuron (v) shows variable responses but not spike-time adaptation.

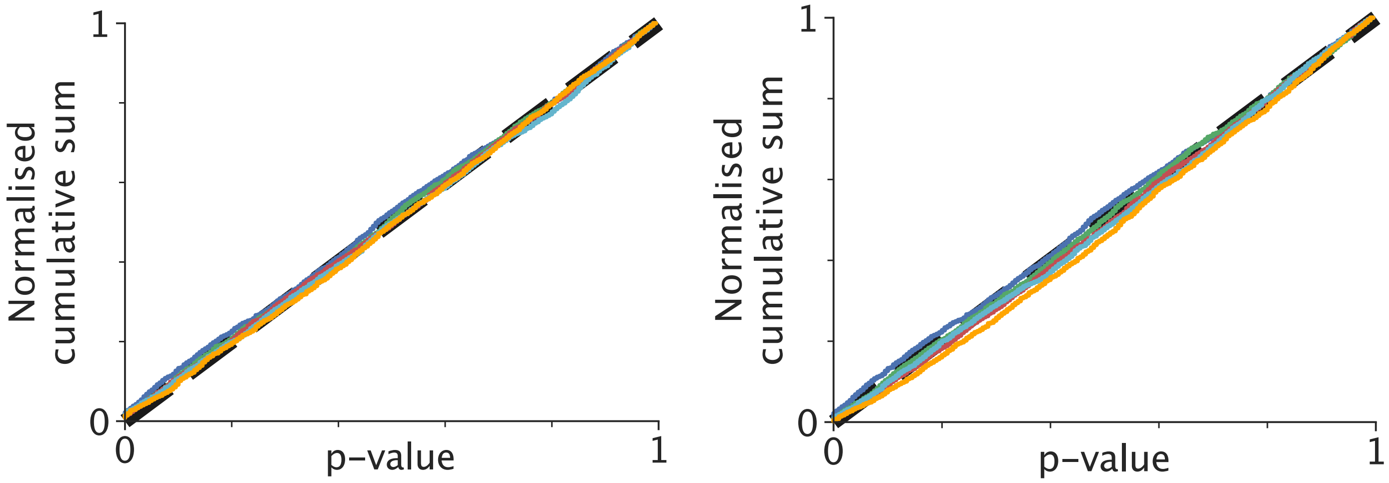

Autocorrelation. Fisher Method (df=4832, N=2416).

Trial-lag	χ^2	p-value
1	5548.9	<0.001
2	5173.6	<0.001
3	5027.3	0.025
4	4954.0	0.108
5	4800.6	0.623

Partial autocorrelation. Fisher Method (df=4832, N=2416).

Trail-lag	χ^2	p-value
2	4806.0	0.602
3	4517.2	>0.999
4	4599.1	0.992
5	4213.8	>0.999

Supplementary Fig. 4. Single-neuron single-stimulus first spike trial-lagged autocorrelations and partial autocorrelations. Trial-lagged autocorrelation and partial autocorrelation analysis allows quantification of further autocorrelative structure in the trial-to-trial sequence of first spike times. Here we consider the first spike times s_t of single neurons on spiking trials t=1,…,T of stimulus condition where T is the number of spiking trials. Trial-lag-n autocorrelations and partial autocorrelations test the correlations and partial autocorrelations between spike times on trials t with spike times on trial t+n.

Plots show the normalized cumulative sums of trial-lag 1-5 autocorrelation (left) and trial-lag (2-5) partial autocorrelation (center) p-values for 2416 single neuron single stimulus combinations. Trial-lagged autocorrelations (left) were above chance for trial-lags 1-3 supportive of general adaptative trends. This is demonstrated by the fact that the cumulative sums are slightly above y=x in the plot, demonstrating non-uniform distributions of p-values. As this is hard to see, accompanying tables show results (χ^2 statistics and p-values) of Fisher-method application to the p-values produced for each trial-lag. This confirms that autocorrelations and are above chance for several values. This is not the case for trial-lagged partial autocorrelations, supporting the presence of general adaptative trends without additional structure in the trial-to-trial sequence of single neuron first spike times.

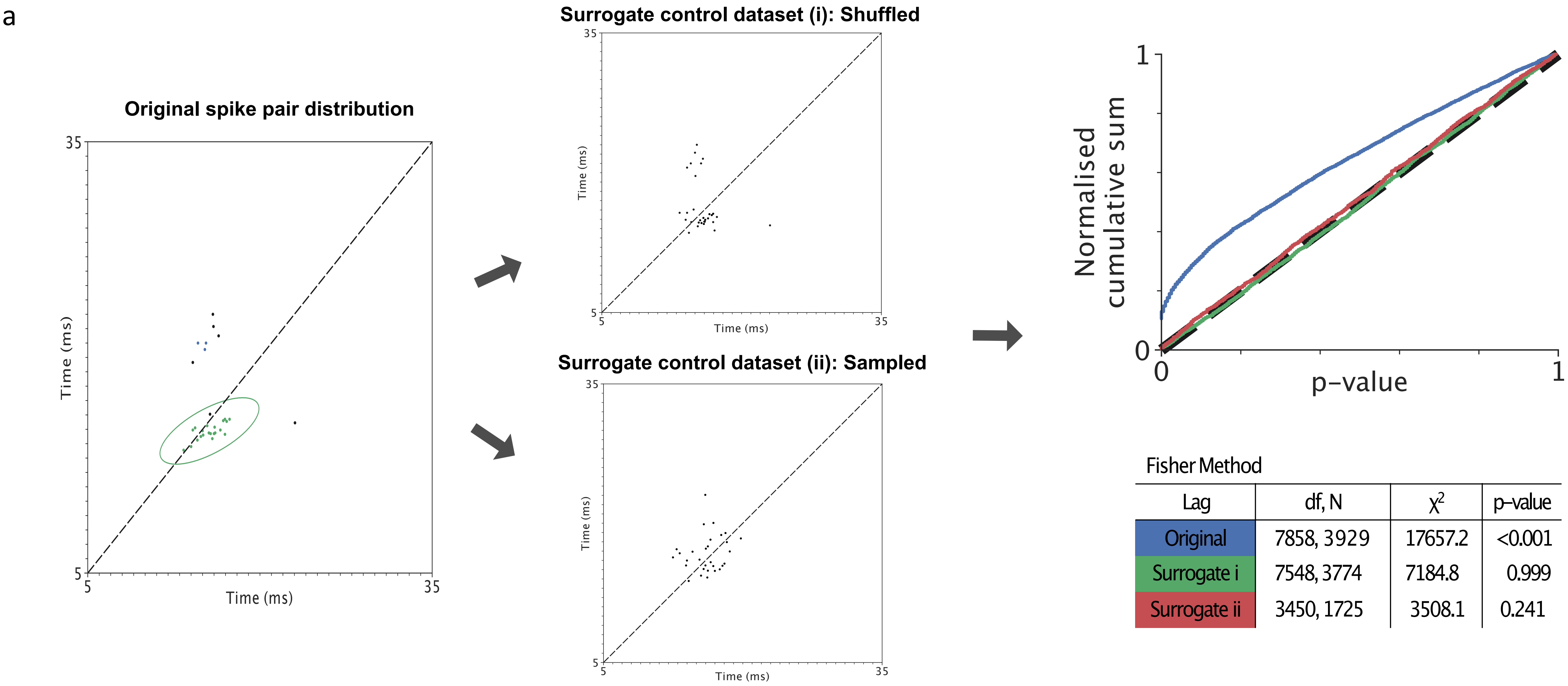

Fisher Method

Lag	df, N	χ^2	p–value
Original	7858, 3929	17657.2	<0.001
Surrogate i	7548, 3774	7184.8	0.999
Surrogate ii	3450, 1725	3508.1	0.241

Supplementary Fig. 5 (1 of 3). Correlation surrogate control. Left: Example of original first spike pair response distribution for repetitions of a single stimulus condition and pair of neurons. Centre: Generated surrogate dataset examples: (i) original response distribution spike times shuffled and (i) spike times sampled from normal distribution with same single neuron standard deviations as original response distribution. Right: cumulative p-value histograms of Stage 1 correlations found by the main clustering algorithm for the original dataset and the two correlation surrogate controls. As described in Methods, figure and table show that correlated clusters were found at chance levels for the two surrogate control datasets.

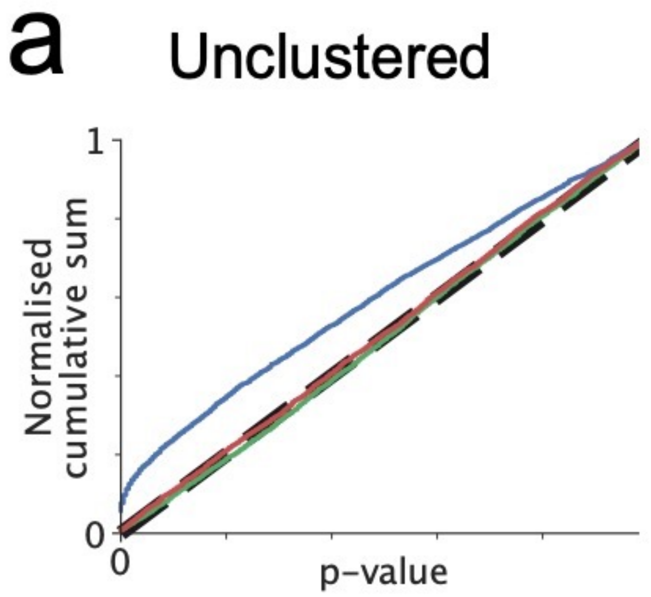

Fisher Method

Lag	df, N	χ^2	p–value
Original	8676, 4338	14863.4	<0.001
Surrogate i	8676, 4338	8356.2	0.993
Surrogate ii	8676, 4338	8721.2	0.364

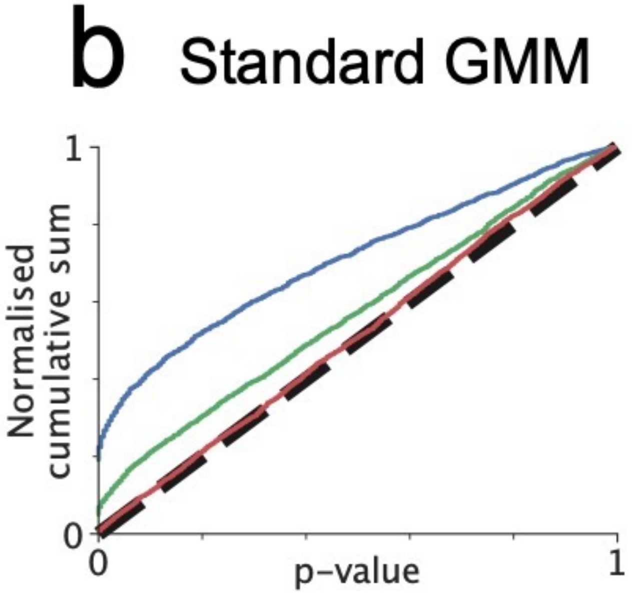

Fisher Method

Lag	df, N	χ^2	p–value
Original	3550, 1775	12142.0	<0.001
Surrogate i	3740, 1870	5830.0	< 0.001
Surrogate ii	6448, 3224	6539.8	0.209

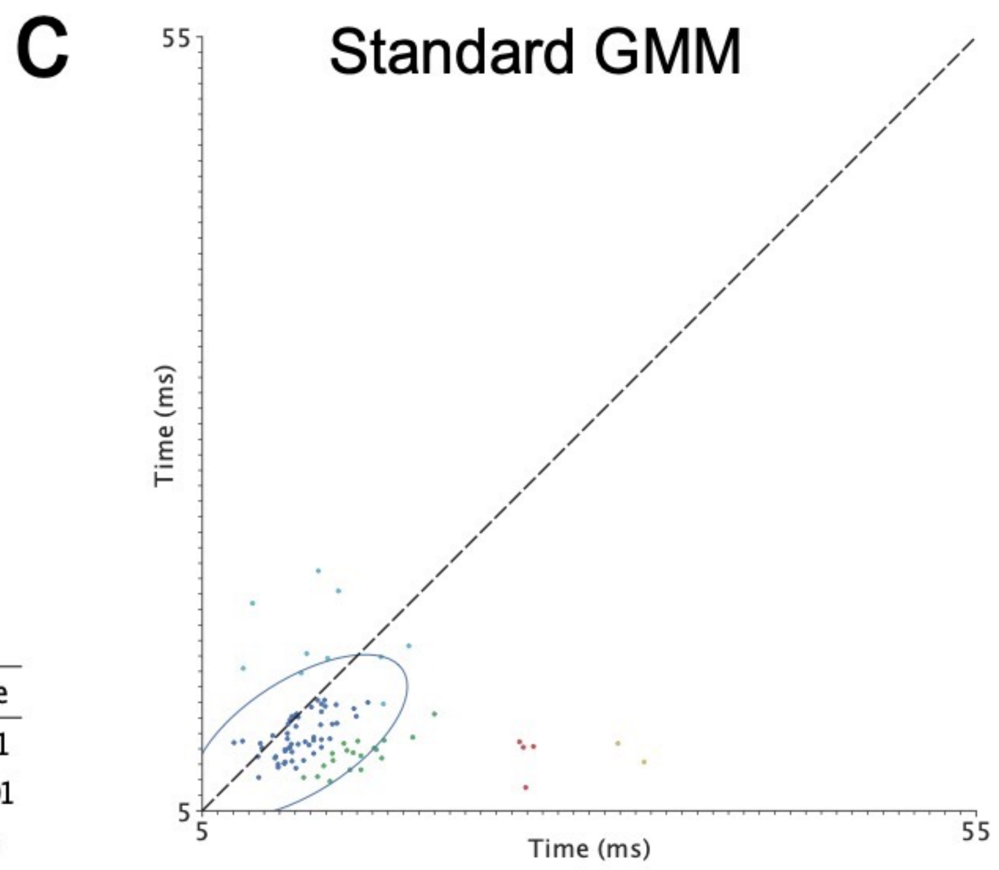

Supplementary Fig. 5 (2 of 3). Correlation surrogate control continued. (a,b) Cumulative p-value histograms of correlations found for the following cases: (a) unclustered and (b) standard GMM. (c) Example detection of stationary correlated cluster (dark blue) in shuffled response distribution through fragmentation by the standard GMM algorithm.

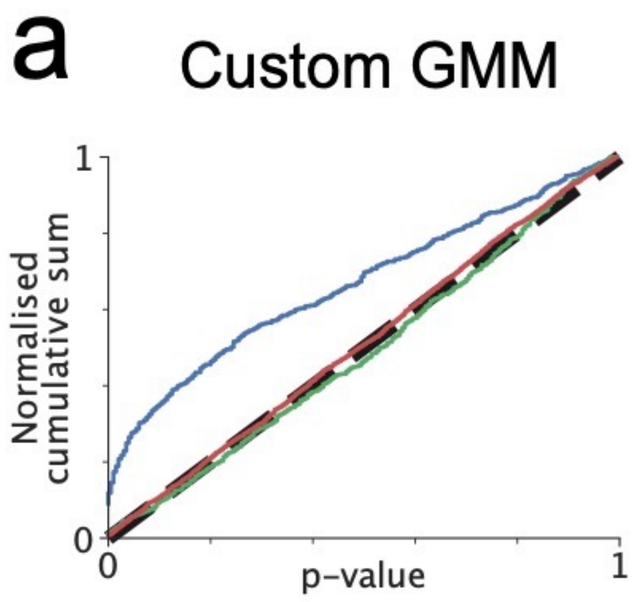

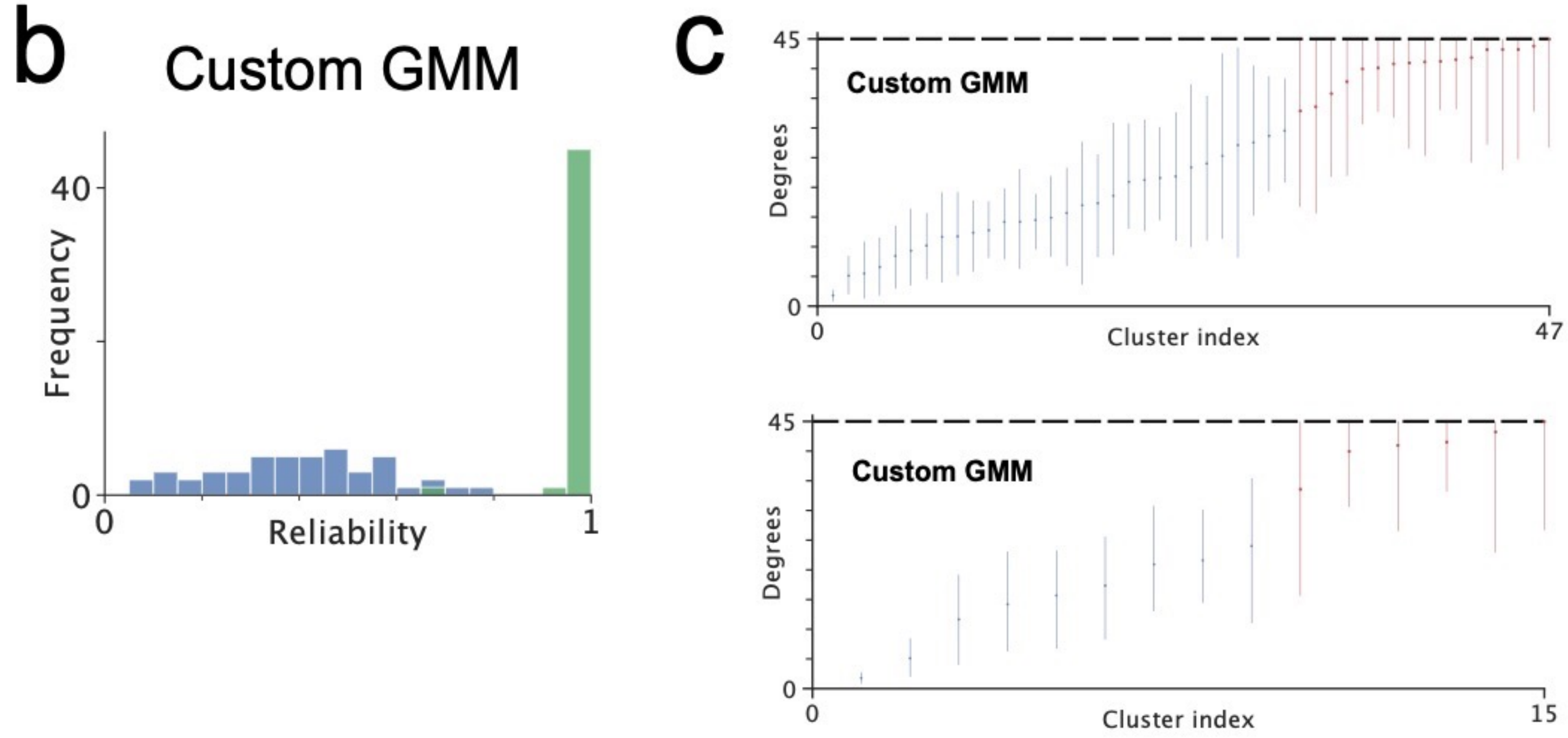

Fisher Method

Lag	df, N	χ^2	p–value
Original	1058, 529	2419.7	<0.001
Surrogate i	1300, 650	1243.5	0.867
Surrogate ii	6384, 3192	6394.6	0.460

Supplementary Fig. 5 (3 of 3). (a) Cumulative p-value histograms of correlations found for custom GMM algorithm. (b) Histogram showing for each Stage 2 cluster found by the custom GMM algorithm the proportion of conjunctive (both neurons spike) trials contributing a cluster sample (blue) and the proportion of stimulus trials contributing a cluster sample (green). (c) Angles of Stage 2 clusters found by the custom GMM algorithm. Mean 45° angles with 95% confidence intervals for positively correlated Stage 2 clusters (top) and stationary Stage 2 clusters (bottom) Blue if significantly greater than 0° (p-value < 0.025) and less than 45° (p-value < 0.025). Red otherwise.

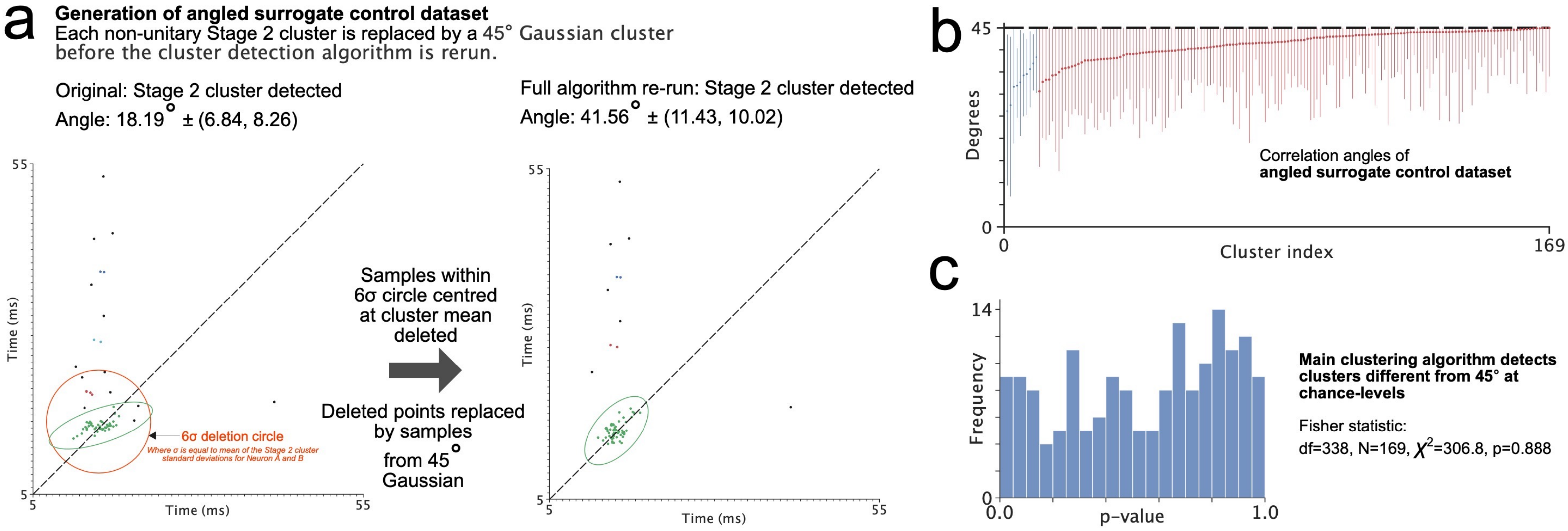

Supplementary Fig. 6 (1 of 3). Angle surrogate control dataset. (a) Left: Generation of angle surrogate control dataset (i) example. Stage 2 cluster detected by main clustering algorithm (green ellipse). Points within 6σ circle of cluster mean (where σ is equal to the mean of the cluster spike time standard deviations of the two neurons) are deleted and replaced by an equal number of samples from a 45° Gaussian ellipse (right). New response distribution added to surrogate control dataset (i). (b) Mean θ_{45} angles with 95% empirical confidence intervals for positively correlated Stage 2 clusters detected in angled surrogate control dataset. Blue if significantly greater than 0° (p-value < 0.025) and less than 45° (p-value < 0.025). Red otherwise. Detection of clusters different from 45° appear to be at chance-levels. (c) Histogram of p-values for correlation angles being different from 45° for Stage 2 clusters detected by the main clustering algorithm for the generated angle surrogate control dataset. Correlation angles different from 45° are not found above chance-levels.

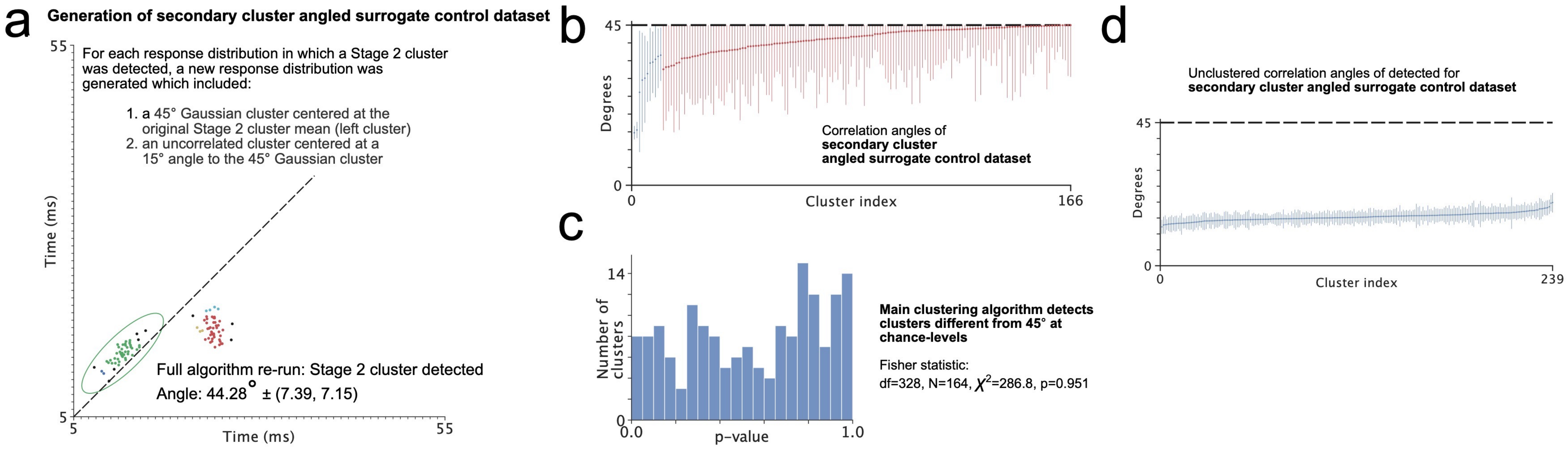

Supplementary Fig. 6 (2 of 3). Secondary cluster correlation angle test. (a) Example surrogate control response distribution generated by drawing samples from a 2D 45° Gaussian ellipse centred at the original Stage 2 cluster mean (left) and a second uncorrelated response distribution with a mean at a 15° angle to the left cluster (right). (b) Mean θ_{45} angles with 95% empirical confidence intervals for positively correlated Stage 2 clusters detected in secondary cluster correlation angle surrogate control dataset. Blue if significantly greater than 0° (p-value < 0.025) and less than 45° (p-value < 0.025). Red otherwise. Detection of clusters different from 45° appear to be at chance-levels. (c) Histogram of p-values for correlation angles being different from 45° for Stage 2 clusters detected by the main clustering algorithm for the generated angle surrogate control dataset. Correlation angles different from 45° are not found above chance-levels. (d) Mean θ_{45} angles with 95% confidence intervals for positively correlated (p<0.005) unclustered response distributions. As expected, the unclustered approach does not detect the angle of the 45° cluster. Instead, correlation angles are found around 15°.

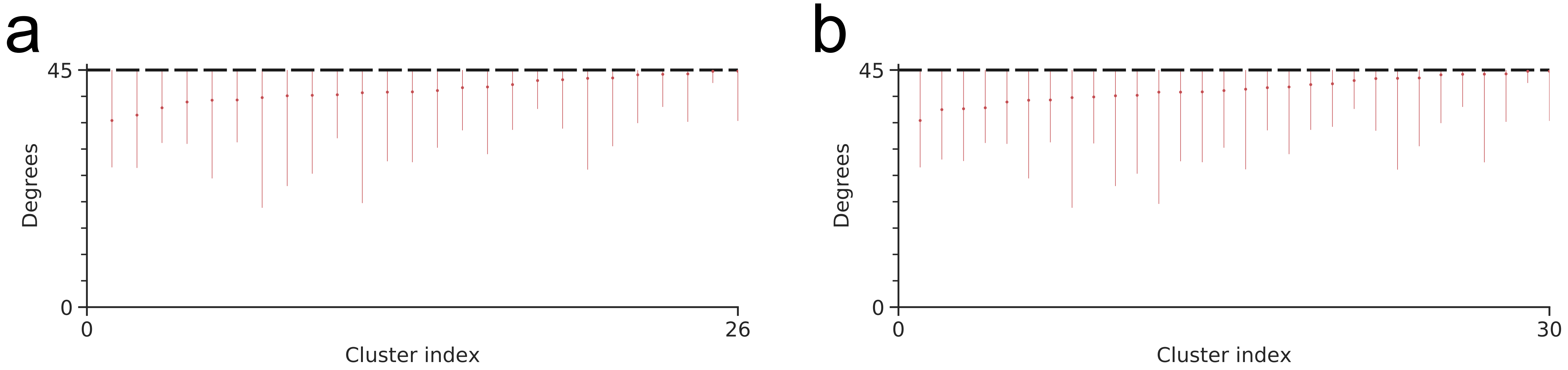

Supplementary Fig. 6 (3 of 3). Angle of Stage 2 clusters detected by Custom GMM algorithm for two angle surrogate control datasets. Mean θ_{45} angles with 95% empirical confidence intervals for positively correlated Stage 2 clusters detected by Custom GMM algorithm in secondary cluster correlation angle surrogate control datasets (generated from response distributions in which Custom GMM algorithm originally detected Stage 2 clusters). Blue if significantly greater than 0° (p-value < 0.025) and less than 45° (p-value < 0.025). Red otherwise. (a) Angle correlation datasets (i). (b) Angle correlation datasets (ii). All detected clusters were not significantly different from 45° for both datasets.

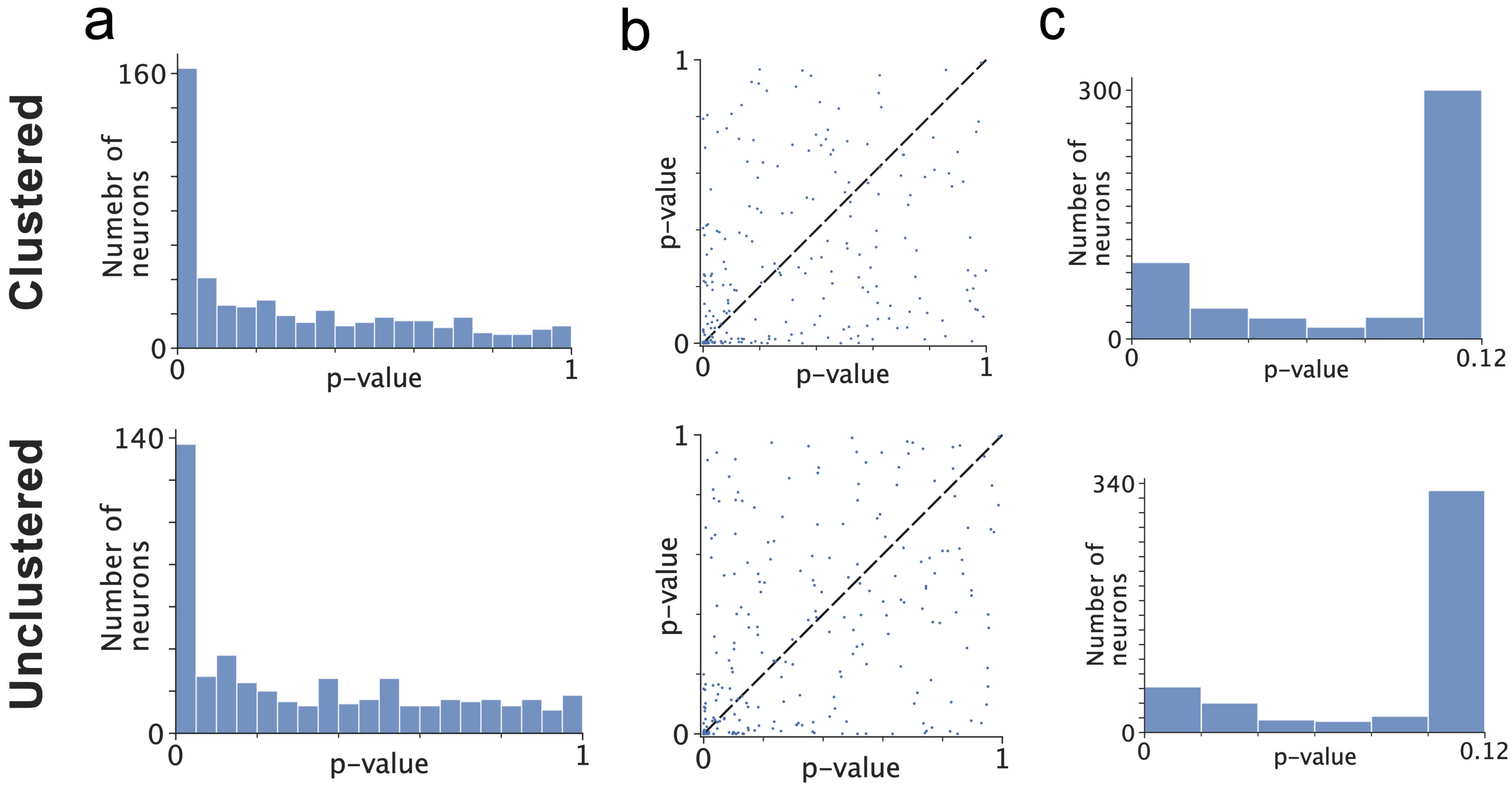

Supplementary Fig. 7. Stationarity of single neurons spike times for Stage 2 clusters (top) and correleated unclustered response distributions (bottom). (a) Correlation p-values of single neuron spike times with trial index. (b) Correlation p-values of single neuron spike times with trial index (neuron A vs neuron B). For some clusters, the cluster spike times of neither neuron were correlated with trial index, whereas for other clusters the spike times of at least one neuron in the cluster pair was correlated with trial index. (c) Histogram of p-values of the **Kwiatkowski–Phillips–Schmidt–Shin trend stationarity test (KPSS)**. The KPSS test was used to test whether the cluster first spike times of single neurons were stationary around a deterministic trend. Single neuron cluster first spike time sequences were determined as not stationary around a deterministic trend if the null hypothesis was rejected ($p < 0.05$). Python `statsmodels' implementation of p-value estimation rounds all p-values > 0.1 to 0.1.

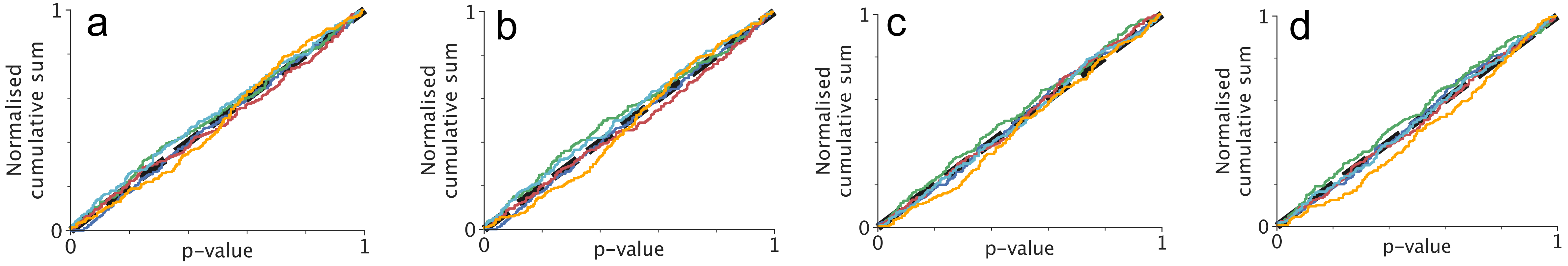

Autocorrelation.
Fisher Method (df=400, N=200).

Trial-lag	χ^2	p-value
1	347.1	0.973
2	417.0	0.269
3	382.3	0.729
4	447.9	0.049
5	367.1	0.880

Partial autocorrelation.
Fisher Method (df=400, N=200).

Trial-lag	χ^2	p-value
2	437.8	0.093
3	369.7	0.859
4	441.2	0.076
5	351.3	0.962

Cross-correlation.
Fisher Method (df=400, N=200).

Trial-lag	χ^2	p-value
1	389.5	0.638
2	423.3	0.203
3	386.1	0.683
4	376.3	0.797
5	329.0	0.996

Partial cross-correlation.
Fisher Method (df=400, N=200).

Trial-lag	χ^2	p-value
2	427.9	0.168
3	374.4	0.816
4	387.2	0.667
5	315.2	>0.999

Supplementary Fig. 8 (1 of 3). Stationary Stage 2 cluster single-neuron single-stimulus first spike (a) autocorrelations, (b) partial autocorrelations, (c) cross-correlation and (d) partial cross-correlation. Autocorrelation, partial autocorrelation, cross-correlation and partial cross-correlation analysis allows quantification of autocorrelative and cross-correlative structure in the trial-to-trial sequence of first spike times and first spike pairs for the 100stationary Stage 2 clusters. Here we consider the first spike times $s_{j,t}$ of neurons j=0,1 on cluster trials t=1,…,T where T is the number of cluster trials for a single cluster. Trial-lag-n autocorrelations and partial autocorrelations test the correlations and partial autocorrelations between single neuron cluster spike times on trials t with spike times on trial t+n. Plots (a) and (b) show the normalized cumulative sums of trial-lag 1-5 autocorrelation and trial-lag (2-5) partial autocorrelation p-values for 200 (=2 * 100 stationary Stage 2 clusters) single neuron single cluster spike time sequences. Trial-lag-n cross-correlations and partial cross-correlations were similarly tested between neuron pair cluster spike times on trials t with spike times on trial t+n.

Autocorrelations, partial autocorrelations, cross-correlations and partial cross-correlations are at chance-levels for trial-lags 1-5. This is demonstrated by the fact that the cumulative sums lie on y=x, demonstrating uniform distributions of p-values. Accompanying tables show results (χ^2 statistics and p-values) of Fisher-method application to the p-values produced for each trial-lag, which confirms that autocorrelations, partial autocorrelations, cross-correlations and partial cross-correlations are not above chance.

Autocorrelation.
Fisher Method (df=420, N=210).

Trial-lag	χ^2	p-value
1	314.3	>0.999
2	417.7	0.523
3	390.5	0.846
4	374.8	0.945
5	371.8	0.956

Partial autocorrelation.
Fisher Method (df=420, N=210).

Trial-lag	χ^2	p-value
2	428.9	0.253
3	377.6	0.932
4	369.6	0.963
5	366.1	0.973

Cross-correlation.
Fisher Method (df=420, N=210).

Trial-lag	χ^2	p-value
1	391.9	0.834
2	443.3	0.228
3	398.9	0.763
4	365.6	0.974
5	391.1	0.840

Partial cross-correlation.
Fisher Method (df=420, N=210).

Trial-lag	χ^2	p-value
2	446.2	0.182
3	388.6	0.862
4	385.3	0.887
5	384.4	0.893

Supplementary Fig. 8 (2 of 3). Stationary correlated response distribution single-neuron single-stimulus first spike (a) autocorrelations, (b) partial autocorrelations, (c) cross-correlation and (d) partial cross-correlation. Same as 1 of 2 but for stationary correlated response distibutions. Normalised p-value histograms not shown.

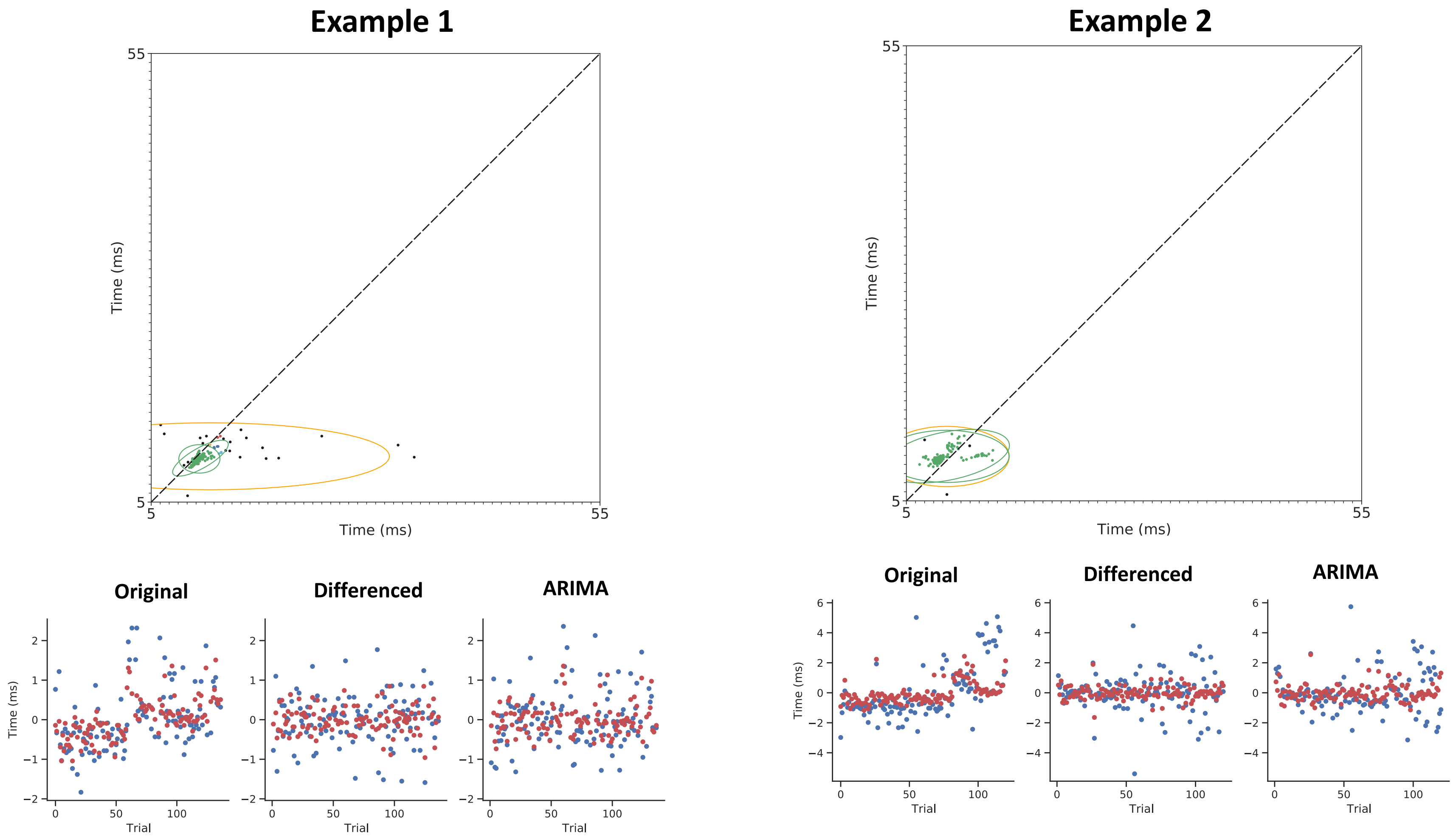

Supp Fig. 8 (3 of 3). Two examples (left and right) of discontinuous and non-monotonic non-stationarity, highlighting the need for differencing over parametric models for the removal of non-stationary trends when testing for non-adaptative pairwise correlations. Top row shows two first spike pair response distributions in which Stage 2 clusters were detected by the main clustering algorithm. Bottom row shows cluster spike times of the two corresponding neurons (red and blue) vs trial index for the original (left), differenced (center) and ARIMA (right) cases. For both of the examples, the original spike times of both neurons show a discontinuity in their relationship with trial index (at around trials 70 and 90 respectively). At these points of discontinuity the latencies of both neurons in both examples become slower, before gradually becoming faster. For the removal of non-stationarities in order to test for pairwise correlations, the non-stationarities of examples such as these could not be modelled with typically used parametric models typically used for adaptative trend removal. Moreover, use of classic parametric models would risk introducing adaptation-independent correlations between the spike times of the two neurons where none existed previously. Alternatively, after the application of differencing and ARIMA, the cluster spike times show no relationship with trial index, and were deemed stationary by the stationarity criteria. This highlights an advantage of differencing based methods over parametric models, for the purposes of this paper.

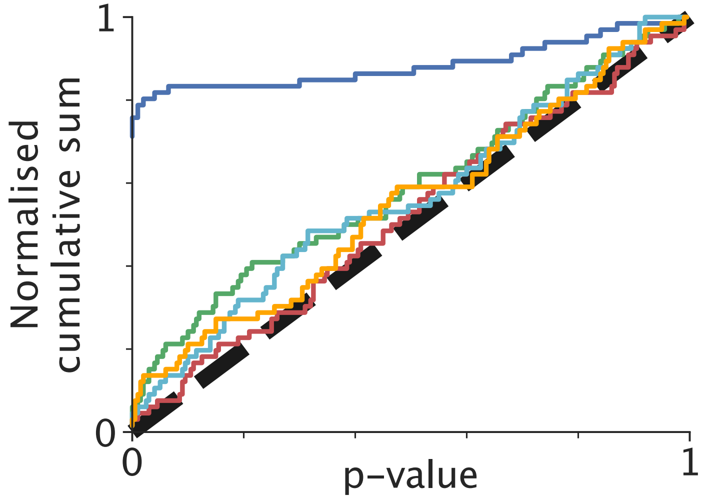

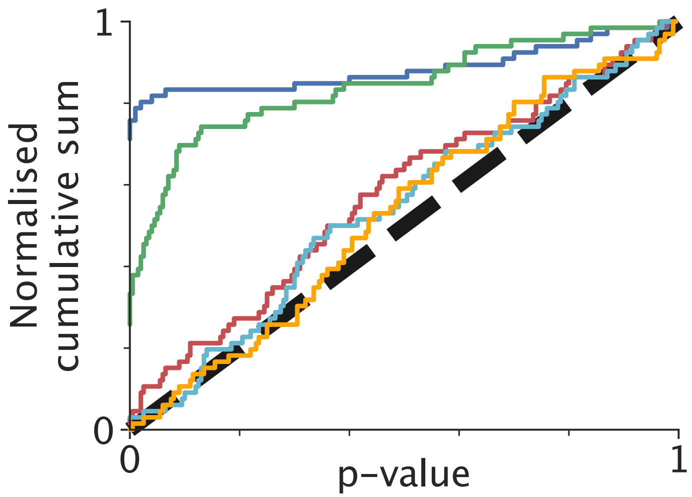

Negative autocorrelation.
Fisher Method (df=132, N=66).

Trial-lag	χ^2	p-value
1	1198.9	<0.001
2	209.2	<0.001
3	143.6	0.230
4	176.8	0.006
5	174.0	0.008

Negative partial autocorrelation.
Fisher Method (df=132, N=66).

Trial-lag	χ^2	p-value
2	504.7	<0.001
3	171.1	0.013
4	146.7	0.181
5	131.7	0.491

Supplementary Fig. 9 (1 of 3). Negative autocorrelations (left) and negative partial autocorrelations (right) introduced by differencing. Plots for criteria fulfilling differenced clusters (with the autocorrelation criterion relaxed; see methods). As with previous autocorrelation plots in Supplementary Fig. 6 but p-values instead represent the probability that single neuron cluster spike times are negatively correlated for the criteria fulfilling differenced Stage 2 clusters. Cumulative sum plots and accompanying tables show that spike times are negatively autocorrelated and negatively partially autocorrelated above chance for several trial-lags, as expected by differencing.

Negative autocorrelation.
Fisher Method (df=148, N=74).

Trial-lag	χ^2	p-value
1	1164.5	<0.001
2	223.5	<0.001
3	188.0	0.015
4	200.5	0.003
5	197.0	0.004

Negative partial autocorrelation.
Fisher Method (df=148, N=74).

Trial-lag	χ^2	p-value
2	573.2	<0.001
3	173.8	0.072
4	156.0	0.311
5	152.3	0.388

Supplementary Fig. 9 (2 of 3). Negative autocorrelations (left) and negative partial autocorrelations (right) introduced by differencing. Same as 1 of 2 but for differenced correlated unclustered response distributions. Normalised p-value histograms not shown.

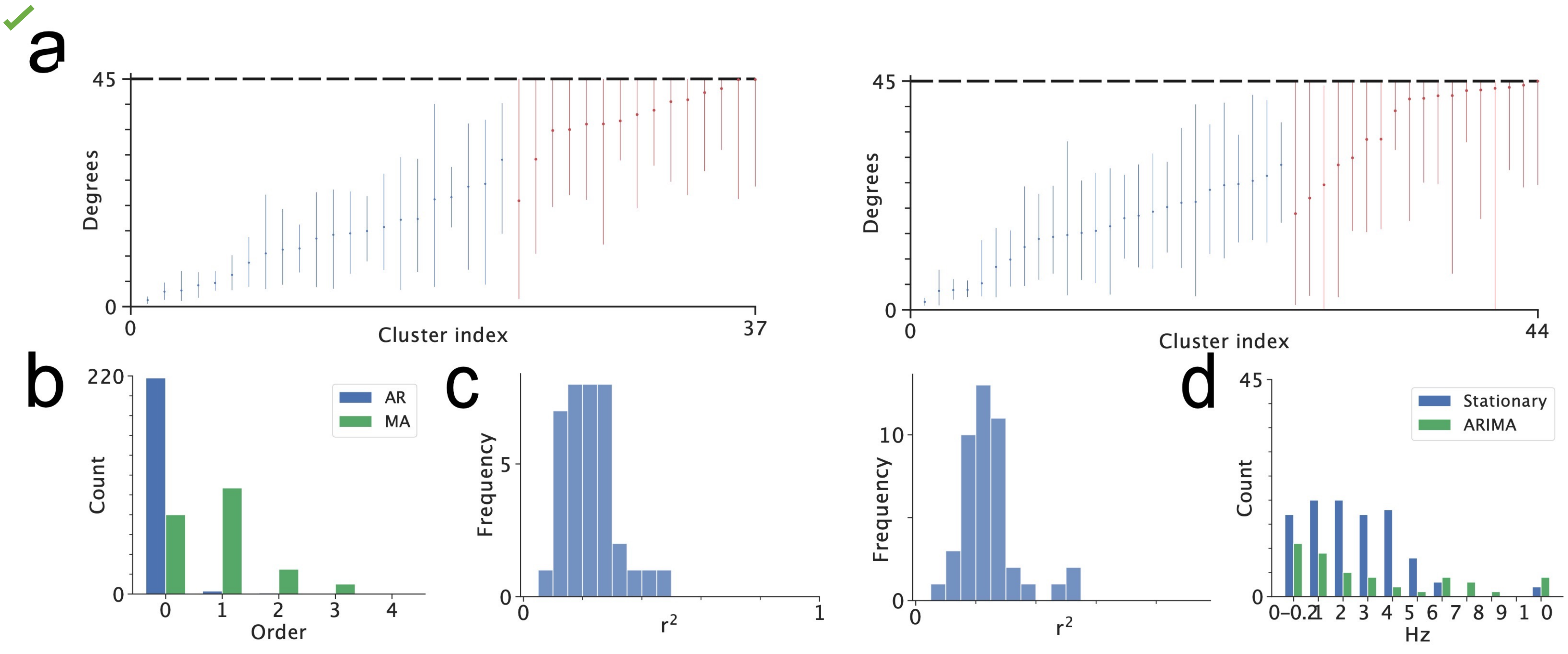

Supp Fig 9 (3 of 3). Time series modelling of correlated unclustered response distributions.
Subfigures a-d are equivalent to 6b-e but for time series modelled correlated unclustered response distributions.

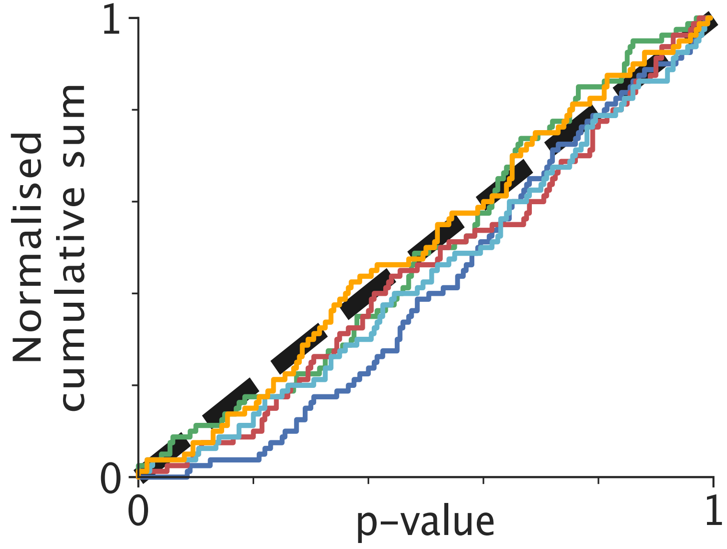

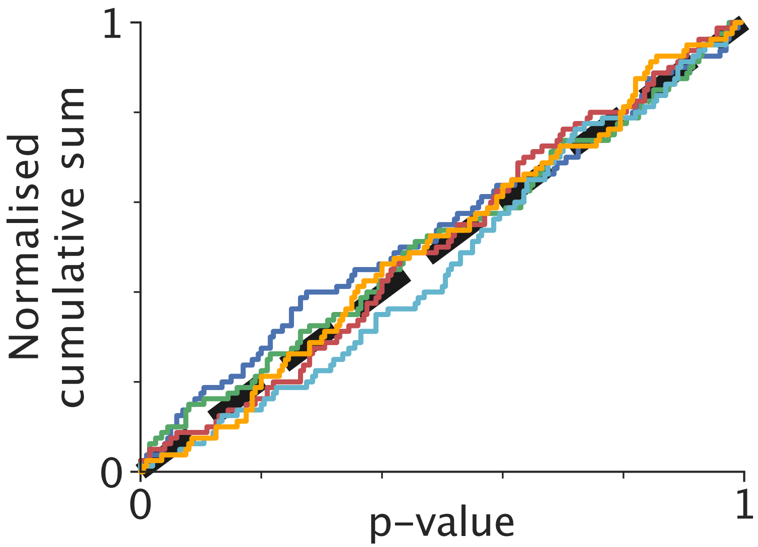

Autocorrelation.
Fisher Method (df=160, N=80).

Trial-lag	χ^2	p-value
1	100.8	>0.999
2	166.8	0.340
3	126.0	0.978
4	135.7	0.918
5	150.0	0.704

Cross-correlation.
Fisher Method (df=160, N=80).

Trial-lag	χ^2	p-value
1	186.1	0.077
2	177.5	0.163
3	163.4	0.410
4	137.3	0.903
5	151.1	0.681

Supplementary Fig. 10 (1 of 2). Autocorrelations (left) and cross-correlations (right) at chance-levels for criteria fulfilling ARIMA modelled Stage 2 clusters. As with Supplementary Fig. 6 (a) and (c) but for the 41 criteria fulfilling ARIMA modelled clusters. Autocorrelations and cross-correlations are also at chance levels confirming that ARIMA modelling successfully removed autocorrelations introduced by differencing and that there is no additional cross-correlative structure between the neurons in ARIMA modelled clusters.

Autocorrelation.
Fisher Method (df=176, N=88).

Trial-lag	χ^2	p-value
1	129.7	0.996
2	150.0	0.923
3	172.1	0.570
4	144.8	0.959
5	164.8	0.717

Cross-correlation.
Fisher Method (df=176, N=88).

Trial-lag	χ^2	p-value
1	181.9	0.365
2	169.0	0.634
3	171.1	0.589
4	160.3	0.796
5	192.7	0.185

Supplementary Fig. 10 (2 of 2). Autocorrelations (left) and cross-correlations (right) at chance-levels for criteria fulfilling ARIMA modelled correlated unclustered response distributions. Same as 1 of 2 but for criteria fulfilling ARIMA modelled correlated unclustered response distributions. Normalised p-value histograms not shown.

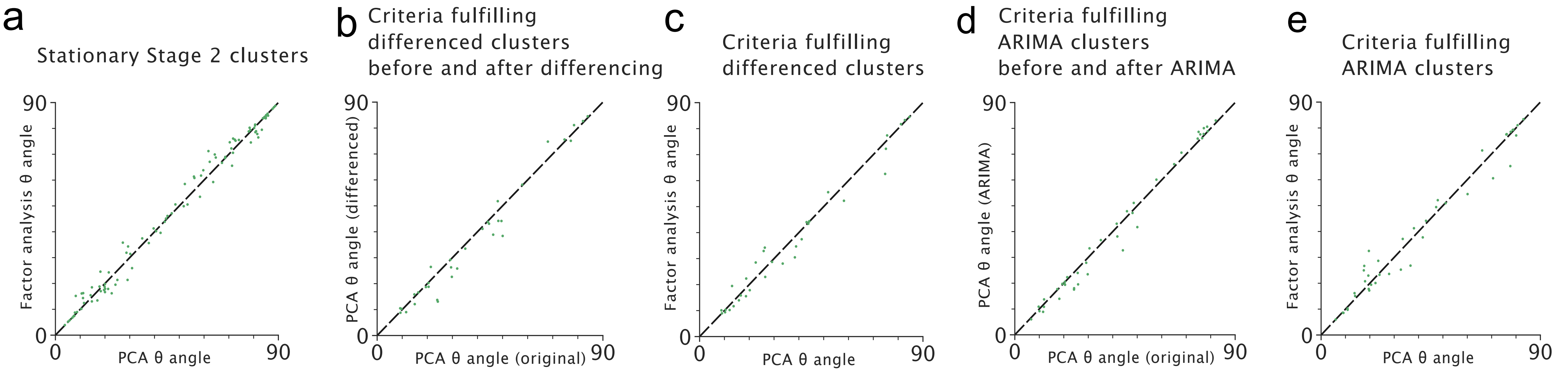

Supplementary Fig. 11 (1 of 2). Cluster angle comparisons. (a) PCA angles vs factor analysis angles for the criteria fulfilling clusters. (b) Original PCA angles vs differenced PCA angles for the criteria fulfilling differenced clusters. (c) PCA angles vs factor analysis angles for the criteria fulfilling differenced clusters. (d) Original PCA angles vs ARIMA PCA angles for the criteria fulfilling ARIMA clusters. (e) PCA angles vs factor analysis angles for the criteria fulfilling ARIMA clusters.

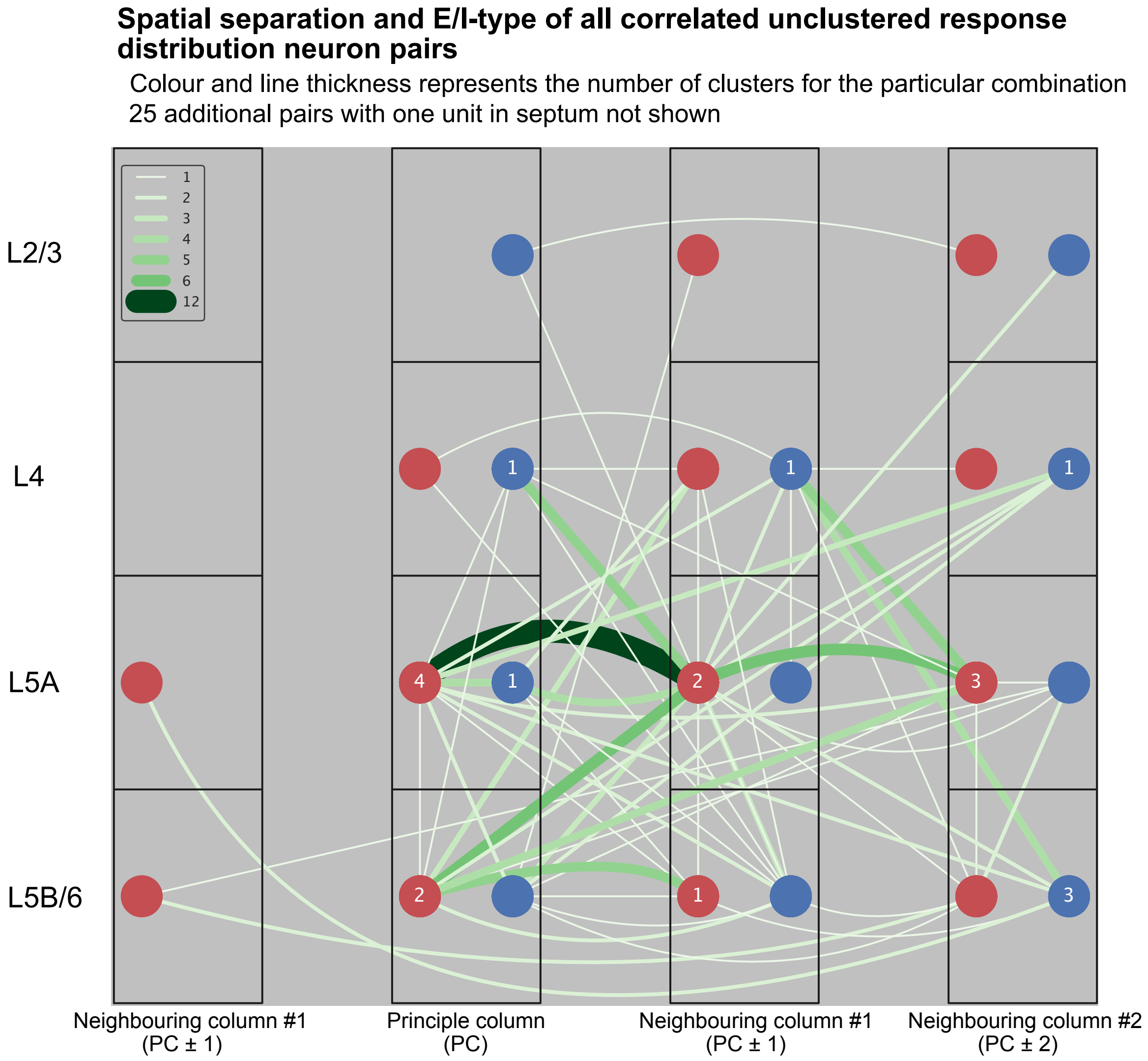

Supplementary Fig. 11 (2 of 2). Same as Figure 7 but for stationary and made-stationary correlated unclsutered response distributions (rather than Stage 2 clusters).

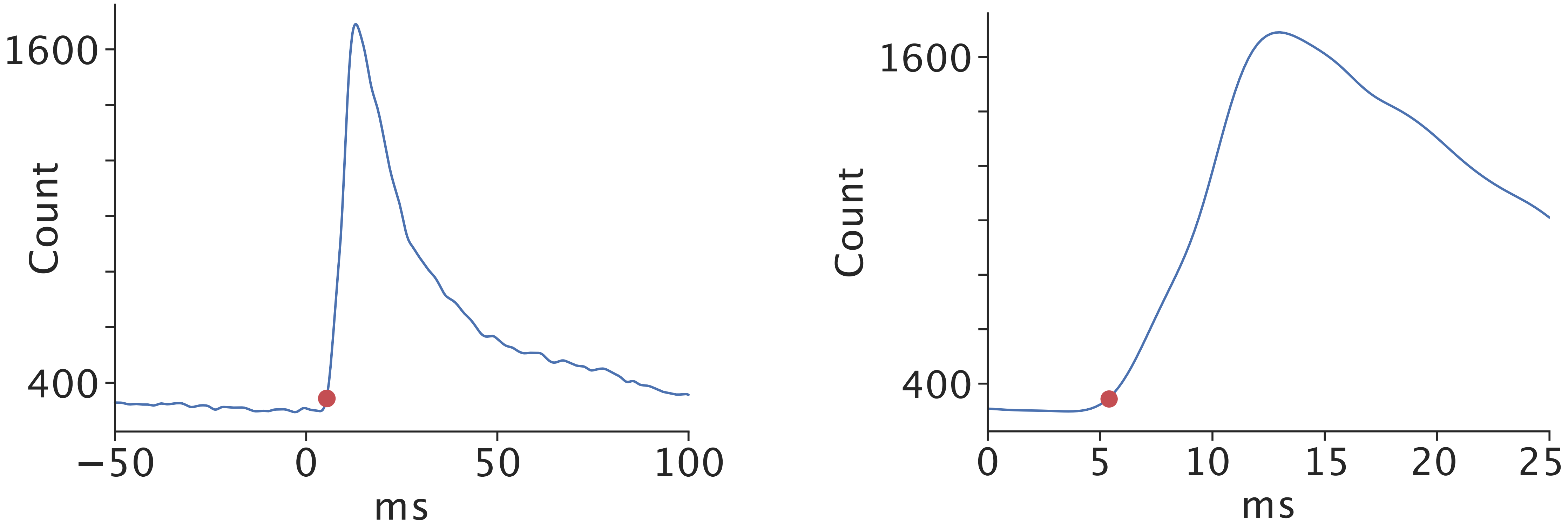

Supplementary Fig. 12. Onset of cortical activation following whisker stimulation. Histogram of single neuron spike times to all stimuli with bins of 0.2 ms and between -50 ms before and +100 ms after stimulus. The histogram was smoothed with a gaussian filter (width s.d. 1 ms). The mean and standard deviation of the smoothed bin counts was calculated for the period before cortical activation onset at 0 ms. The cortical activation onset spiking threshold was determined as this mean value + 3 standard deviations. Cortical onset was determined as the time point when the smoothed histogram went above this threshold (red dot). The left and right figures show the same smoothed histogram between -50 and +100 ms (left) and 0 and +25 ms (right).

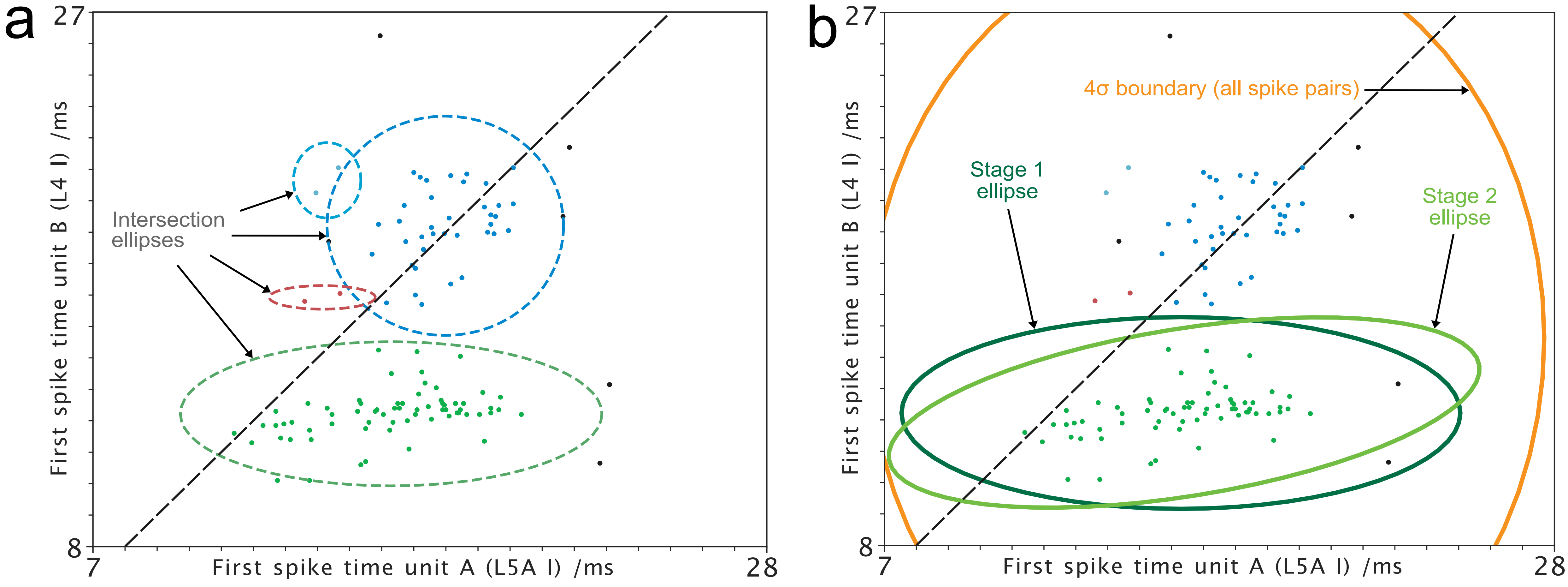

Supplementary Fig. 13. Example clustering of sample first spike response distribution for two neurons (L5AI, L4I) for single epsilon value over a 50ms window following cortical onset (1 outlier out of view). (a) Point colours illustrate DBSCAN clustering (epsilon = 1.35). Black points are outliers. Dashed ellipses represent 3σ intersection ellipse for each DBSCAN cluster. Setting the DBSCAN `minimum number of elements in a cluster' parameter to 2 increased the cluster quality control, as intersection ellipses were created for 2 element clusters rather than assigning them as outliers. (b) Dark green flat ellipse illustrates 4σ confidence interval of Stage 1 cluster. Light green angled ellipse illustrates 4σ confidence interval of Stage 2 cluster. Orange ellipse illustrates 4σ ellipse of all spike pairs under the assumption of independence and normality.

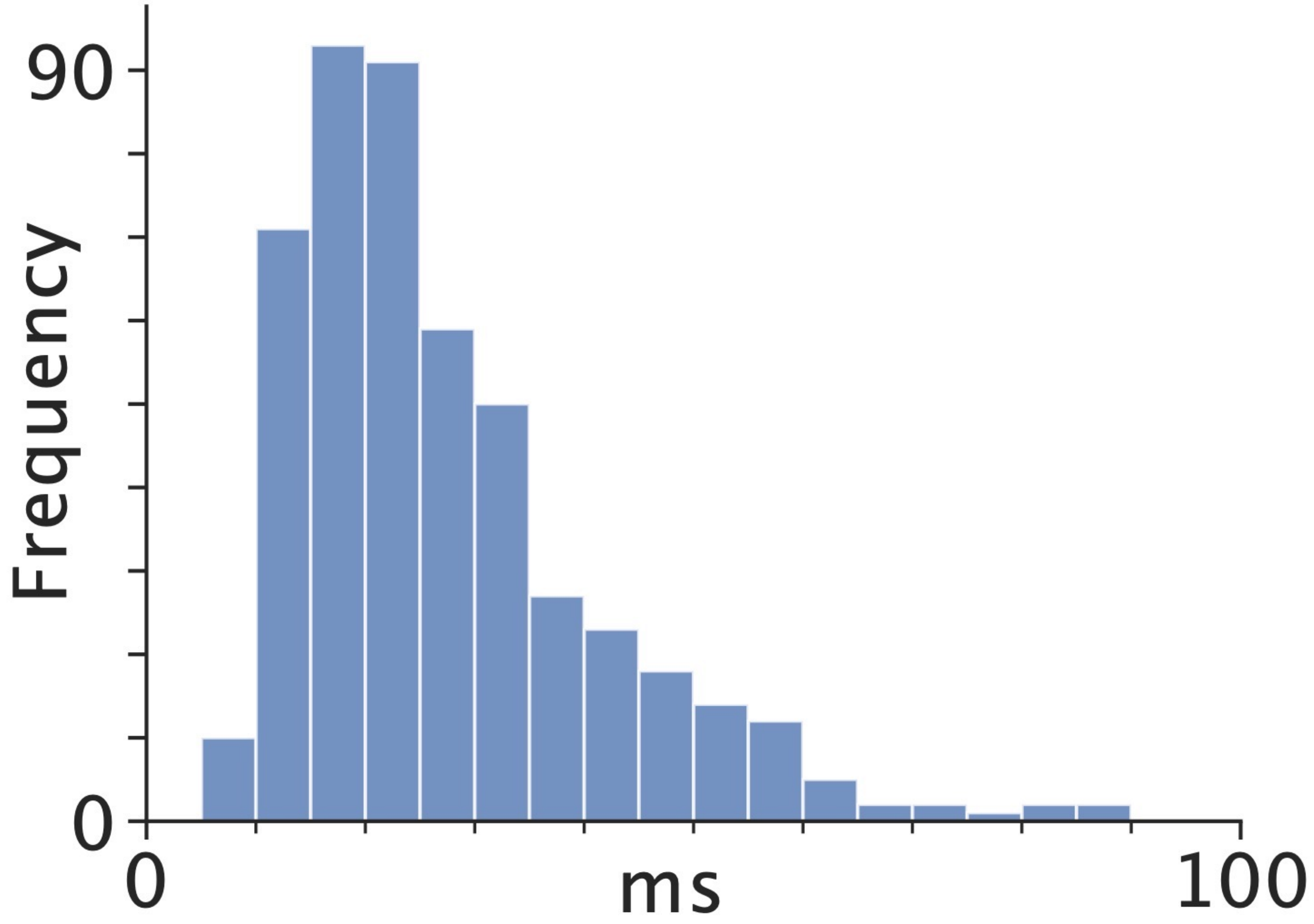

Supplementary Fig. 14. Cluster end points. Histogram of Stage 2 cluster end points (mean + 4σ) for Stage 2 clusters detected over 100ms following cortical activation onset.
